# Unravelling the electrochemical double layer by direct probing of the solid/liquid interface

**DOI:** 10.1038/ncomms12695

**Published:** 2016-08-31

**Authors:** Marco Favaro, Beomgyun Jeong, Philip N. Ross, Junko Yano, Zahid Hussain, Zhi Liu, Ethan J. Crumlin

**Affiliations:** 1 Advanced Light Source, Lawrence Berkeley National Laboratory, One Cyclotron Road, Berkeley, California 94720, USA; 2 Joint Center for Artificial Photosynthesis, Lawrence Berkeley National Laboratory, One Cyclotron Road, Berkeley, California 94720, USA; 3 Chemical Sciences Division, Lawrence Berkeley National Laboratory, One Cyclotron Road, Berkeley, California 94720, USA; 4 Ertl Center for Electrochemistry and Catalysis, School of Environmental Science and Engineering, Gwangju Institute of Science and Technology, Gwangju 500-712, Republic of Korea; 5 Center for Advanced X-ray Science, Gwangju Institute of Science and Technology, Gwangju 500-712, Republic of Korea; 6 Materials Sciences Division, Lawrence Berkeley National Laboratory, One Cyclotron Road, Berkeley, California 94720, USA; 7 Molecular Biophysics and Integrated Bioimaging Division, Lawrence Berkeley National Laboratory, One Cyclotron Road, Berkeley, California 94720, USA; 8 State Key Laboratory of Functional Materials for Informatics, Shanghai Institute of Microsystem and Information Technology, Chinese Academy of Sciences, Shanghai 200050, People's Republic of China; 9 Division of Photon Science and Condensed Matter Physics, School of Physical Science and Technology, ShanghaiTech University, Shanghai 200031, China; 10 Joint Center for Energy Storage Research, Lawrence Berkeley National Laboratory, One Cyclotron Road, Berkeley, California 94720, USA

## Abstract

The electrochemical double layer plays a critical role in electrochemical processes. Whilst there have been many theoretical models predicting structural and electrical organization of the electrochemical double layer, the experimental verification of these models has been challenging due to the limitations of available experimental techniques. The induced potential drop in the electrolyte has never been directly observed and verified experimentally, to the best of our knowledge. In this study, we report the direct probing of the potential drop as well as the potential of zero charge by means of ambient pressure X-ray photoelectron spectroscopy performed under polarization conditions. By analyzing the spectra of the solvent (water) and a spectator neutral molecule with numerical simulations of the electric field, we discern the shape of the electrochemical double layer profile. In addition, we determine how the electrochemical double layer changes as a function of both the electrolyte concentration and applied potential.

## Abstract (web summary)

The electrochemical double layer is a key concept in chemistry, but its properties are hard to probe experimentally. Here, the authors use ambient pressure X-ray photoelectron spectroscopy to probe the electrochemical double layer potential profile at the solid/liquid interface, under polarization conditions.

## Graphical abstract

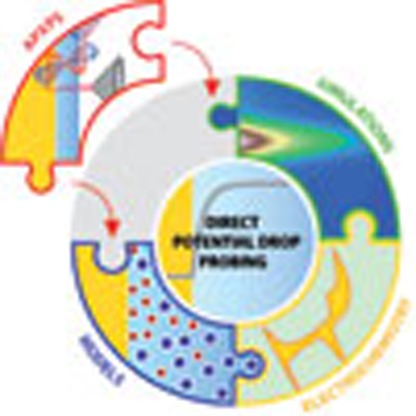

The electrochemical double layer (EDL), originally conceived by Hermann von Helmholtz in the nineteenth century, constitutes a key concept in modern electrochemistry of electrified interfaces^1^,^2^,^3^. Researchers^4^,^5^ have produced many refinements of Helmholtz's early theoretical work, eventually leading to the well-known Gouy–Chapman^6^ (GC) and Gouy–Chapman–Stern^6^ (GCS) modelling of the EDL. Subsequent studies based on electrochemical measurements have been performed by Gurney^7^, Grahame^4^,^8^, Parsons^5^,^8^, Bockris^9^ and most recently by Trasatti^10^,^11^ and Conway^12^, with the aim of addressing common limitations of the three aforementioned models, such as the lack of detailed knowledge of the specific interactions between the ionic species in the bulk solution as well as within the electrified layers near the metal electrode, which are considered purely Coulombic in the GC or GCS models^13^.

The properties of the interface formed by a charged electrode surface immersed in an electrolyte governs the charge transfer processes through the interface itself, thus influencing the electrochemical responses of the electrode/electrolyte system^14^,^15^. These concepts and models together serve as the foundation of modern electrochemistry. A comprehensive investigation of the EDL structure and associated charge transfer processes constitutes an essential step towards understanding and improvement of a variety of electrochemical processes, such as electrocatalysis, electrochemical energy storage, ion transport through biological membranes and corrosion^4^,^5^,^14^,^15^,^16^.

Various electrochemical approaches^5^,^8^,^17^,^18^ have been utilized to experimentally investigate EDL properties, such as the study of the electrocapillarity^4^ and the influence of surface adsorbates^19^ with different binding strengths^5^,^18^. Investigations of the EDL have re-emerged on account of new spectroscopy techniques such as optical-vibrational spectroscopies based on infrared absorption^20^ and Raman scattering^21^,^22^. Since the pioneering studies of Kolb, Wagner and Ross on immersed electrodes, several groups have employed X-ray photoelectron spectroscopy (XPS) measurements not under polarization conditions, showing that it is possible to study the specific adsorption of ions at the Inner Helmholtz Plane (IHP)^23^,^24^,^25^,^26^,^27^,^28^. Similarly, Siretanu *et al*. investigated the ionic structure within the Stern layer by studying the corresponding force field with scanning probe microscopies^29^. Synchrotron X-ray characterization techniques have also been utilized^27^,^28^,^29^,^30^,^31^,^32^: Bedzyk *et al*.^30^ used X-ray standing waves (SW) to show that the cation distribution in the liquid phase obeys the GCS model, while Velasco-Velez *et al*.^31^, using X-ray absorption spectroscopy, reported on the strength of the hydrogen bonds in water molecules at the IHP, highlighting significant differences compared with bulk water.

However, despite these initial studies on the EDL structure^18^,^23^,^24^,^25^,^26^,^27^,^28^,^29^,^30^, the information of the electrical potential profile at the solid/liquid-electrified interface, particularly as a function of the applied potential, was still elusive.

In this work, we report the direct probing of the potential drop (PD) as well as the potential of zero charge (PZC) by means of ambient pressure X-ray photoelectron spectroscopy (APXPS) performed under polarization conditions. By analyzing the spectra of the solvent (water) and a spectator neutral molecule with numerical simulations of the electric field, we discern the shape of the EDL profile. In addition, we determine how the EDL changes as a function of both the electrolyte concentration and applied potential.

## Results

### APXPS probing of the potential drop

**Figure 1:**
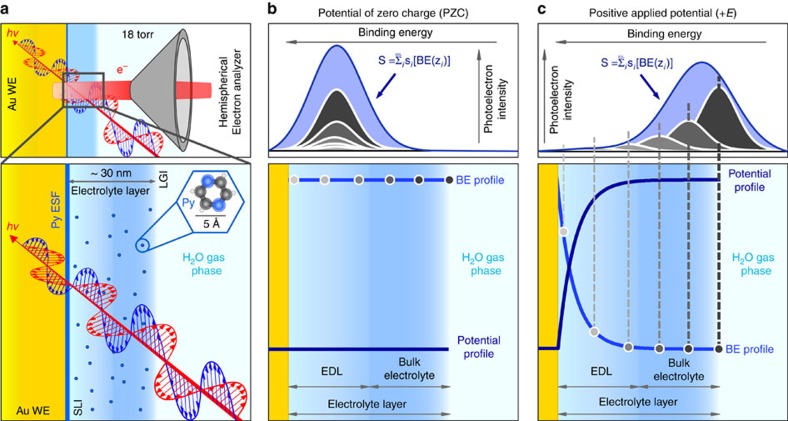
Schematization of the electrochemical double layer probing by APXPS under polarization conditions. (**a**) EDL probing using a gold polycrystalline working electrode (WE) in KOH 0.4 mM aqueous solution (*d*_EDL_=15.2 nm) containing 1.0 M pyrazine (SLI, solid/liquid interface, LGI, liquid/gas interface, and Py ESF, pyrazine at the electrode surface). (**b**,**c**) Schematic representing the spectral broadening of the core level of the elements belonging to the liquid phase, passing from the potential of zero charge (PZC) to a positive potential applied to the WE, respectively. The function *S* represents the detected liquid phase related-core level, which is given by the convolution of the binding energy (BE)-shifted spectra of the molecules in solution (*s*_*j*_[BE(*z*_*j*_)]) as a function of their position within the potential drop with respect to the WE surface (*z*_*j*_).

We performed APXPS^32^,^33^ under polarization conditions at the working electrode (WE)/liquid electrolyte interface^34^,^35^,^36^,^37^,^38^ using an excitation energy of 4.0 keV^39^,^40^,^41^. The advantage of using ‘tender' X-rays (2.0–8.0 keV) relies on the fact that the inelastic mean free path in an aqueous electrolyte and for escaping photoelectrons with a kinetic energy >3,500 eV is ∼10 nm. This provides the ability to directly probe the electrical potential experienced by ions and molecules^35^,^36^,^37^,^38^,^39^,^40^,^41^,^42^ in a 10–30 nm-thick electrolyte layer^39^,^40^,^41^,^42^, a dimension that coincides with the EDL thickness for a dilute solution (see Supplementary Fig. 1 and the schematization reported in Fig. 1a).

In this work, an electrolyte liquid layer (comprised of an aqueous 0.1–80.0 mM potassium hydroxide (KOH) with 1.0 M pyrazine (Py) solution) having a thickness of about 30 nm (Supplementary Fig. 1) was successfully created on a polycrystalline gold electrode by the ‘dip and pull' method^39^,^40^,^41^,^42^ (Supplementary Fig. 2) and characterized within the double-layer potential region ranging from −450 to +650 mV (versus Ag/AgCl/Cl^−^_(sat.)_ for all potentials unless otherwise stated; the electrochemical responses are reported in Supplementary Fig. 3). In addition to its element-specific and chemical specific nature, XPS provides information about the local potentials of the atoms that undergo the photoionization process^43^. By measuring the core-level binding energy (BE) shift of the elements present in the liquid layer as a function of the applied potential at the electrode, it is possible to determine local potentials^39^,^40^,^41^,^42^ within the liquid layer (the energy diagram of the solid/liquid/gas interface is schematically reported in Supplementary Fig. 4). The key spectroscopic parameter to probe the potentials within the EDL in this study is the full-width at half-maximum (FWHM) of the core-level peaks from elements within the electrolyte, which undergo a broadening as the PD in the EDL changes.

The observed spectral broadening for elements in the water (O) and pyrazine (N) molecules, the latter introduced as an independent molecular probe and uniformly distributed within the liquid phase^44^ (Fig. 1a), is caused by the electric field in the double layer originated by the charge on the electrode surface. At the PZC, for which no net charge density is present at the electrode surface (that is, no net PD is present in solution), the detected core level (O 1*s* from water or N 1*s* from pyrazine) shows a reduced spectral broadening, as schematically described in Fig. 1b. The schematization of the spectral broadening as a function of the applied potential, when a PD is present in the electrolyte, for a gold electrode in a 0.4 mM KOH aqueous solution (creating an EDL thickness equal to 15.2 nm) containing 1.0 M pyrazine is shown in Fig. 1c. In this experimental system, water and pyrazine molecules experience different electric potentials as function of its *z*-position within the PD^45^ (distance normal to the electrode surface, see Fig. 1c); consequently, the corresponding escaping O 1*s* or N 1*s* photoelectrons are characterized by different apparent binding energies (referenced to the Fermi level of the WE). The final result of the convolution of the single shifted O 1*s* and/or N 1*s* core-level photoelectron peaks (Fig. 1c) leads to the experimentally observed spectral broadening (an increase in the FWHM), which becomes broader the larger the electrode potential for a given EDL thickness (that is, the higher the PD within the double layer, compared with the PZC conditions, Fig. 1b).

**Figure 2:**
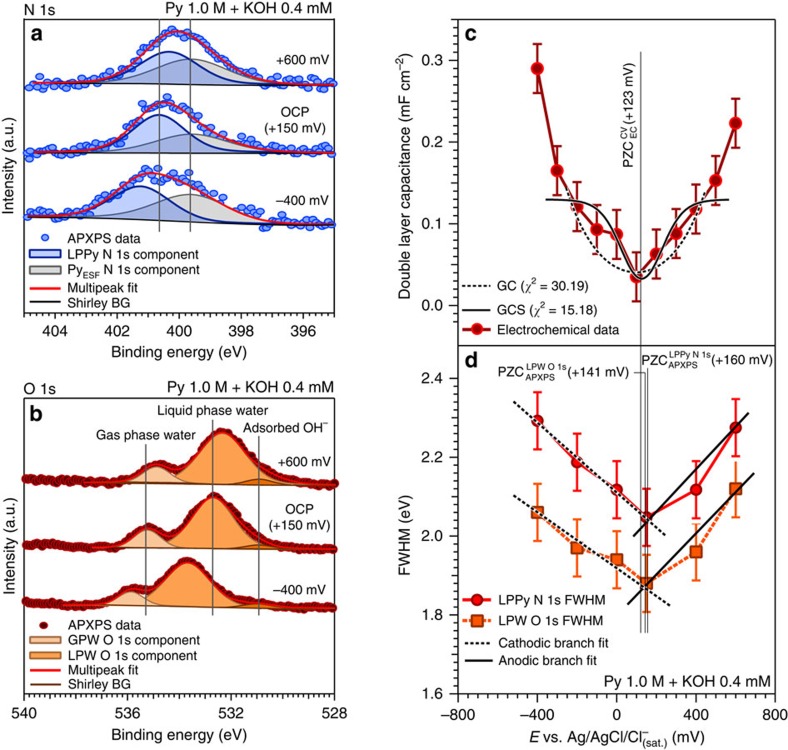
Electrochemical double layer probing from the spectral broadening of pyrazine and water core levels. (**a**,**b**) Representative intensity-normalized N 1*s* and O 1*s* core-level peaks, respectively, acquired at different applied potentials to the working electrode (WE) in a KOH 0.4 mM aqueous solution (*d*_EDL_=15.2 nm) containing 1.0 M pyrazine (OCP, open circuit potential, APXPS, ambient pressure X-ray photoelectron spectroscopy, LPPy, liquid phase pyrazine, GPW, gas phase water, LPW, liquid phase water, and BG, background) and (**c**) double-layer capacitance (obtained from electrochemical characterization) as a function of the applied potential. The double layer capacitance trend has been fitted within a range of 400 mV centred on the PZC, by using both Gouy–Chapman (GC) and Gouy–Chapman–Stern (GCS) models^6^,^18^; (**d**) LPPy N 1*s* and LPW O 1*s* full-width at half-maximum (FWHM) trends as a function of the applied potential within the EDL region (APXPS, ambient pressure X-ray photoelectron spectroscopy, CV, cyclic voltammetry, and EC, electrochemistry). The error bars were determined via repeated measurements of the FWHM of a given core level, propagated with the experimental spectral resolution (see Methods section).

The intensity-normalized N 1*s* and O 1*s* core-level peaks are shown in Fig. 2a,b (with an applied vertical offset for clarity) as a function of the applied potential, from −400 to +600 mV within the double-layer capacitance region (see Supplementary Fig. 5 for all spectra). The N 1*s* raw spectra possess two different identifiable components: the low BE feature centred at 399.7 eV is attributed to pyrazine molecules at the electrode/electrolyte interface (which we will refer to as pyrazine at the electrode surface, Py_ESF_), while the high BE component corresponds to the solvated pyrazine molecules present in the liquid, hereafter labelled as liquid phase pyrazine, LPPy N 1*s* (further details regarding the Py_ESF_ characterization are reported in Supplementary Figs 6–9 and Supplementary Note 1). Similarly, the O 1*s* spectra were de-convoluted by using three different chemically shifted components attributed to gas phase water (GPW), liquid phase water (LPW) and adsorbed hydroxyls^34^,^36^ at the solid/liquid interface (from highest to lowest BE). From these data, we can observe that the Py_ESF_ and the adsorbed hydroxyls do not shift in BE with the applied potential, meaning that the corresponding species are electrically connected to the grounded gold WE (see the energy diagram reported in Supplementary Fig. 4 for further details). Most importantly, as the potential is applied with respect to the open circuit potential (OCP), the LPPy N 1*s* and LPW O 1*s* peak positions shift in BE and undergo a FWHM broadening, as schematically reported in Fig. 1b,c. The observed spectral broadening and BE shifts for the components within the liquid phase (that is, within the EDL) constitute the essential results of this work.

Although the linear trend of the BEs as a function of the applied potential inherently carries information about the PD within the EDL, the peak FWHM trend allows an easier and direct comparison with the double-layer capacitance trend (derived from electrochemical investigations), as shown in Fig. 2c. In this manner, the core-level peak FWHM represents the direct link between spectroscopy and electrochemistry.

The PZC, defined as the observed potential at which no net charge is present on the electrode surface and no PD is present in the EDL can be determined both electrochemically (Fig. 2c) using the cyclic voltammetry (CV) method and from the APXPS spectra (Fig. 2d).

The trend of the double-layer capacitance reported in Fig. 2c as a function of the applied potential shows the characteristic V-shape, where the minimum point determines the PZC^31^,^46^. Under the experimental conditions, we found that the PZC (+123 mV) is very close to the OCP value (+150 mV). Interestingly, the spectral broadening of the LPPy N 1*s* component (Fig. 2d) also shows the same V-shape dependence as a function of the applied potential, with the minimum reached at the OCP (which has been demonstrated to be close to the PZC value, under the conditions of the present experiment). To extract the PZC from the spectroscopy data of both core levels, we have superimposed two fitted straight lines to the anodic and cathodic branch of the FWHM trend of the LPPy N 1*s* and LPW O 1*s* components as a function of the applied potential, as reported in Fig. 2d. The PZC determined in this manner is equal to +160 and +141 mV by using the LPPy N 1*s* and LPW O 1*s* component, respectively, versus +123 mV derived from the CV method. This agreement reflects the linkage between the double-layer capacitance and the trend of the peak FWHM values, thus establishing a clear correlation between an electrochemical property of the EDL and a pure spectroscopic feature. It is noteworthy to report that the findings discussed above are valid also in case of a pyrazine free electrolyte, since the direct probing of EDL can be performed, as reported in Fig. 2, through the analysis of the LPW O 1*s* core-level peak. More details are provided in Supplementary Fig. 5 and Supplementary Note 2.

### Comparison between experimental data and numerical simulations

**Figure 3:**
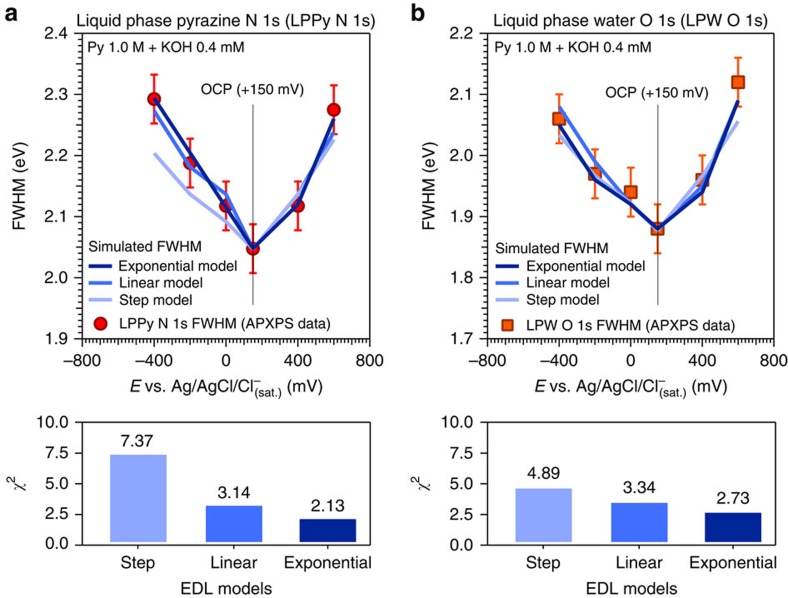
Extrapolation of the potential drop functional dependency from the spectral broadening of pyrazine and water core levels. Comparison between the experimental and simulated FWHM trends using the liquid phase pyrazine (LPPy) N 1*s* (**a**) and the liquid phase water (LPW) O 1*s* (**b**) peaks, as function of the applied potentials and for a KOH concentration of 0.4 mM (*d*_EDL_=15.2 nm) (OCP, open circuit potential and FWHM, full-width at half-maximum); an evaluation of the statistical goodness of the fit through a chi-square analysis (*χ*^2^) is provided for the different electrochemical double layer (EDL) models and core levels used for the EDL probing (LPPy N 1*s* and LPW O 1*s*, respectively). The error bars were determined via repeated measurements of the FWHM of a given core level, propagated with the experimental spectral resolution (see Methods section).

Additionally, we observed that the FWHM trends as a function of the applied potential implicitly carries the functional dependency fingerprint of the PD (Fig. 3).

To access this information, we superimposed the LPPy N 1*s* and the LPW O 1*s* FWHM experimental data (Fig. 3a,b) with numerical simulations results modelling the PD within the double layer as a step, a linear and an exponential function (where the exponential function resembles the GC or GCS models; see Supplementary Fig. 10).

The step and linear PD models were selected to test the fit to our data as well as our ability to distinguish the shape of the EDL structure, and thus do not represent a modern theory for the EDL structure. We then utilized a statistically significant ‘goodness of fit' parameter (chi-square (*χ*^*2*^)) to discern which PD model (step, linear and exponential) best fits the data. This approach provides the ability to objectively evaluate the agreement between the data and models for all applied potentials and electrolyte concentrations (that is, EDL thicknesses, *d*_EDL_). As well documented by Fig. 3a,b and the corresponding *χ*^2^ values, for each core level the best fit between the experimental FWHM trends and the simulated data is consistently realized when the PD within the EDL is modelled as an exponential function. This is in agreement with the accepted GCS modelling of the EDL for diluted solutions and low polarizations (with respect to the PZC)^6^,^18^.

Currently, the depth resolution that characterizes the APXPS measurements does not allow us the ability to discriminate between the GC and the GCS model. Due to the exponential decay of the photoelectron intensity, the spectral components localized at the IHP do not significantly contribute to the summation over all the initial states. Future experimental and instrumental modifications are being planned to help improve this limitation. Moreover, the utilization of the SW-APXPS approach to provide a higher degree of spatial resolution near the electrode surface will be explored^42^,^47^, in particular towards the study of surface adsorbates, ion-specific adsorptions and charge transfers at the IHP, and their interplay in the faradaic processes occurring at the electrified interface.

**Figure 4:**
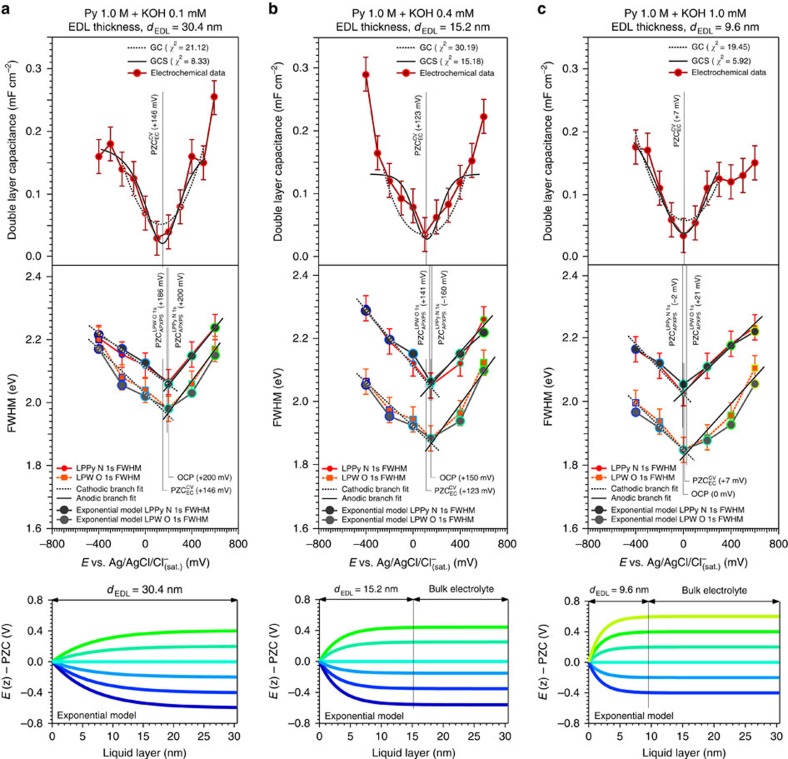
Electrochemical double layer probing and potential drop evolution as a function of the applied potential and electrolyte concentration. Potential of zero charge (PZC) determination and comparison between the experimental and simulated spectral full-width at half-maximum (FWHM) trends, as function of the applied potentials (*E*) and for an aqueous electrolyte containing 1.0 M pyrazine and a KOH concentration equal to 0.1 mM (**a**), 0.4 mM (**b**) and 1.0 mM (**c**), determining an EDL thickness (*d*_EDL_) of 30.4, 15.2 and 9.6 nm, respectively. The bottom part of the figure panels reports the simulated PD profiles for the different EDL thicknesses, as a function of the applied potential (LPPy, liquid phase pyrazine, LPW, liquid phase water, OCP, open circuit potential, CV, cyclic voltammetry, EC, electrochemistry, GC, Gouy–Chapman model, GCS: Gouy–Chapman–Stern model, and APXPS, ambient pressure X-ray photoelectron spectroscopy). The error bars were determined via repeated measurements of the FWHM of a given core level, propagated with the experimental spectral resolution (see Methods section).

To get more insights into the EDL structure as a function of the electrolyte concentration, we have modulated the EDL thickness (*d*_EDL_) by gradually increasing the electrolyte concentration, passing from 0.1 mM (*d*_EDL_=30.4 nm), 0.4 mM (*d*_EDL_=15.2 nm) up to 1.0 mM (*d*_EDL_=9.6 nm), while keeping fixed the concentration of the probe molecule in solution (1.0 M pyrazine). The N 1*s*, O 1*s* and Au 4*f* data for each investigated electrolyte concentration are reported in Supplementary Fig. 11. Further details are also reported in Supplementary Note 3. By using the aforementioned linear fitting procedure applied to the experimental FWHM trends as a function of the applied potential, we have determined the PZC values and described the evolution of the exponential potential profile directly from the spectroscopy data shown in Fig. 4. The PZC values determined in this way (by using both LPPy N 1*s* and LPW O 1*s* core-level peaks) are in line with those derived from the CV method. In addition, the comparison between the experimental data and the simulations based on the envelope function approach has demonstrated that the best match is consistently achieved (for different electrolyte concentrations and using the spectral broadening of both LPPy N 1*s* and LPW O 1*s* core levels) when the PD is modelled with an exponential function, as predicted by the GCS model (the *χ*^*2*^ values for the three different models and the investigated conditions are reported in Supplementary Table 1). However, with the current signal-to-noise ratio, the definitive discrimination between an exponential and a linear shape for the PD is challenging to achieve.

## Discussion

Future improvements of the experimental set-up will enhance spectral resolution and signal-to-noise ratio, which will improve our ability to discern the fine features of the PD functional dependency carried by the core-level spectral broadening. The simulations of the PD profiles, similarly to what has been experimentally performed, have been generated as a function of the applied potential for different electrolyte concentrations (that is, for different EDL thicknesses). The comparison between the experimental and simulated FWHM provides the ability to directly access and visualize the PD within the EDL that leads to the observed spectral broadening. The resulting simulated exponential potential profiles are reported in Fig. 4. This possibility paves the way toward a new type of EDL (and its inherent properties such as the PD) investigations under real electrochemical and photoelectrochemical reactions.

Finally, for KOH concentrations higher than those shown in Fig. 4, the de-convolution of O 1*s* spectra from LPW and N 1*s* spectra from the pyrazine probe molecules showed that the contribution from the molecules within the bulk electrolyte dominates, leading to a rigid spectral shift and only to a weaker broadening of the core levels as a function of the applied potential.

A specific example for a KOH concentration of 80 mM is reported in Supplementary Fig. 12. This result is consistent with a thinner EDL that can be expected from an increased ionic strength of the supporting electrolyte. There is not enough signal from the molecules in the EDL to be resolved from the total O 1*s* and N 1*s* photoelectron intensity.

Therefore, there is a concentration limit for this technique to discern the electrical potential profile within the EDL. The schematization reported in Supplementary Fig. 13 illustrates this limitation. However, future experimental and instrumental modifications previously mentioned (that is, SW-APXPS) will allow us to overcome these current limitations^42^,^47^.

In summary, we have experimentally probed the potential distribution in the EDL at an electrified solid/liquid interface by means of APXPS performed under polarization conditions. The method has general applicability and is not restricted to one specific class of solids or liquids. We have shown that the FWHM trend of the core-level peaks of electrolyte as a function of the applied potential is strongly correlated to the double-layer capacitance trend. We have also demonstrated that the PZC obtained from spectroscopy is in line with the electrochemically-derived values, providing a synergistic correlation between electrochemistry and photoelectron spectroscopy. Exploiting the element-specificity and potential-sensitivity of photoelectron spectroscopy, we have directly probed the PD within the EDL by studying the broadening of the core-level photoelectron spectra of the elements belonging to the liquid phase, as a function of the applied potential. Furthermore, by using numerical simulations, we have shown that our method provides the ability to identify the functional dependency that describes the PD within the double layer, providing a spectroscopic support for the current electrified interface theories. A schematic representation of our combined experimental and simulation-based approach is reported in Supplementary Fig. 14. The coupling between our APXPS performed under polarization conditions with the use of a solvent- or an independent probe-related core level, sets a paradigm for probing the properties of the EDL under realistic conditions. The current results may open the door for studying the EDL dynamics in more complicated systems, such as redox reactions at photoactive semiconductor interfaces and in non-aqueous electrolytes, to name a few (see Supplementary Note 1). The knowledge gained from this method may thus impact a wide range of scientific fields, including electrochemical conversion and storage of energy, environmental sciences and biology^14^,^15^,^16^.

## Methods

### Synchrotron light source and APXPS setup

Beamline 9.3.1 at Advanced Light Source (ALS, Lawrence Berkeley National Laboratory) is equipped with a bending magnet and a Si (111) double crystal monochromator having a total energy range between 2.0 and 8.0 keV (‘tender' X-ray range)^39^. The analyzer (R4000 HiPP-2, Scienta) pass energy was set to 200 eV, using a step of 100 meV and a dwell time of 300 ms. Under these conditions, the total resolution (source and analyzer) was equal to 250 meV at room temperature (r.t.). The measurements were taken using a photon energy of 4.0 keV at r.t. and in normal emission, at a pressure in the experimental chamber ranging from 16 and 18 torr (see following section), while the detection stage in the analyser was under high vacuum conditions (∼10^−7^ torr). The calibration of the BE scale was carried out using the Au 4*f* photoelectron peak as reference (4*f*_7/2_ BE=84.0 eV), from a clean gold polycrystalline surface.

Charging of the electrolyte^48^ was not observed in this study during the APXPS measurements: Supplementary Fig. 15 shows both N 1*s* and O 1*s* core levels acquired at the OCP after 3 h of X-ray exposure (that is, initially collected at the beginning and again at the end of an experiment, where the EDL was probed as a function of the applied potential in an aqueous KOH 0.4 mM solution containing 1.0 M pyrazine). The X-ray dose absorbed by the electrolyte does not lead to shift in the BE nor in the OCP values.

All the fit reported in this work have been carried out using a Doniach-Šunjić shape for the Au 4*f* photoelectron peak, whereas a symmetrical Voigt function (*G*/*L* ratio ranging from 85/15 to 75/25) was used to fit the N 1*s* and the O 1*s* photoelectron peaks (after Shirley background subtraction). During the fitting procedure, the Shirley background was optimized as well, together with the spectral components, increasing in this manner the precision and reliability of the fitting procedure^49^,^50^,^51^. The *χ*^2^ minimization was ensured by the use of a nonlinear least squares routine, with increased stability over simplex minimization^50^. In this work, we have used the FWHM for the quantification of the spectral broadening.

### Electrochemical measurements and ‘Dip and Pull' method

The chemicals here employed were high purity reagents and they were used as received without further operations. MilliQ water (DI, *ρ*=18.2 MΩ cm) was used as solvent, while KOH (99.99%, Aldrich) and pyrazine (Py, Aldrich, 99.99%) were used as supporting electrolyte and molecular probe, respectively.

The working and counter electrodes (Au and Pt polycrystalline foils respectively, Aldrich) were polished to a mirror finish with silicon carbide papers of decreasing grain size (Struers, grit: 2,400 and 4,000). The samples were then cleaned by two cycles of ultrasonic treatment in a mixture of MilliQ water/ethanol (Aldrich, 1:1) for 10 min. A third ultrasonic cleaning was then conducted in pure MilliQ water for 15 min, followed by thoroughly rinsing and drying in N_2_ stream.

Once the three electrodes were mounted on the manipulator^39^ (Supplementary Fig. 2) in the APXPS endstation, an electrochemical cleaning procedure^23^ was conducted by cycling the potential between −1.1 and +1.4 (versus the Ag/AgCl/Cl^−^_(sat)_ reference electrode) for a total number of 100 cycles (using a Biologic SP 300 potentiostat/galvanostat). The cleaning procedure was stopped after reaching the OCP of the cell from the cathodic side (that is, after HER, to start the EDL probing on a cleaned metal surface^23^).

Before its introduction into the experimental chamber, the electrolyte (KOH 0.1, 0.4, 1.0 and 80.0 mM aqueous solution) was outgassed for at least 30 min at low pressure (around 10 torr) in a dedicated off-line chamber. Then, once the manipulator and the outgassed electrolyte were placed into the experimental chamber, the pressure was carefully lowered down to the water vapour pressure (between 16 and 20 torr, at r.t.); finally, the dip and pull procedure was carried out to obtain a stable and conductive nanometric-thick electrolyte layer on the WE surface^39^,^40^,^41^,^42^.

As regards the PZC determination, each double-layer capacitance value was determined at a pressure between 16 and 20 torr (at r.t.), integrating the area of the CV curves within a 20 mV potential window centred at each chosen potential (within the double-layer region of the electrode/electrolyte assembly)^31^. The voltammetric measurements were conducted at a scan rate of 5 mV s^−1^.

### Numerical PD simulations

To rationalize the results obtained experimentally and to prove that using the APXPS approach it is possible to describe the functional dependency of the PD, a series of numerical simulations of the experimental data have been developed (both for the LPW O 1*s* and LPPy N 1*s* photoelectron peaks), modelling the PD within the EDL as a step, a linear or an exponential function (the latter resembling the GC or GCS models^6^,^18^). The first two models were considered to test the ability of distinguishing between different PD functional dependencies. The core algorithm was developed in Igor (C-oriented) language. An example of the performed simulations is reported in Supplementary Fig. 10.

Simulated N 1*s* or O 1*s* photoelectron peaks have been generated by using the envelope function-derived method^52^, by summing the different spectral contributions within the diffuse layer (each one shifted according to the potential profile) and in the bulk electrolyte (rigidly shifted because of the shift of the liquid phase with the applied potential, see energy diagram reported in Supplementary Fig. 4). Then, by performing the summation over all the shifted N 1*s* (or O 1*s*) spectral components with a coefficient determined by the exponential dumping of the photoelectron intensity (Beer–Lambert law), it is possible to finally obtain a simulated spectrum that can be compared with the experimental data. The envelope function *S*(*BE*) at the base of the calculation, describing the final convolution of the simulated photoelectron peak as reported in equation 1:


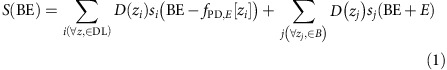


*s*_*i,j*_(BE) and *D(z*_*i,j*_) are the single shifted spectral component and the corresponding coefficient, respectively; the starting spectra *s*(BE) were modelled as a Voigt function (*V*=*G*/*L* ratio equal to 3:1) with FWHM and BE determined by fitting the N 1*s* and O 1*s* photoelectron peaks acquired under APXPS conditions from a ‘bulk' 1.0 M Py aqueous solution. The measurement was performed studying a thick drop of solution hanging from the WE, to rule out interfacial contribution and obtain the O 1*s* and N 1*s* photoelectron peaks exclusively from water and solvated pyrazine molecules in the bulk electrolyte, respectively. *f*_PD,*E*_[*z*_*i*_] is the shift applied to the BE of the spectral components within the PD; it depends on the applied potential *E* and on the particular mathematical model describing the PD. The spectral components within the bulk electrolyte are instead fully shifted by the applied potential *E*. The two integers *i* and *j* vary over the PD within the EDL and the bulk region of the electrolyte layer (*B*), respectively. The simulations were conducted probing both the EDL and the bulk electrolyte every 0.1 nm.

A D'Alembert test was performed to find the minimum probing step that allows full convergence of the summation reported in equation (1), resulting equal to 0.4 nm. The algorithm developed for the calculation of the simulated spectra as described by equation (1) is characterized by a good stability also for a significant number (*n*) of total elements in the summation; the complexity class of the algorithm belongs to *O(n)*.

The *D*_*i,j*_ coefficients that damps the intensity of the single summation elements are calculated according to the Beer–Lambert law (equation (2))


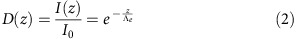


*Λ*_*e*_ is the inelastic mean free path determined through the Tanuma–Powell–Penn algorithm^53^, since the single scattering albedo is negligible for excitation energies above 2.0 keV^54^,^55^; *Λ*_*e*_ (for an incoming photon energy of 4.0 keV and a 1.0 M pyrazine aqueous solution as attenuating medium) is equal to 8.8 nm and to 9.3 nm for an N 1*s* (BE=400.6 eV) and an O 1*s* (BE=532.8 eV) escaping photoelectron, respectively.

### Data availability

The data that support the findings of this study are available from the corresponding author upon request.

## Additional information

**How to cite this article:** Favaro, M. *et al*. Unravelling the electrochemical double layer by direct probing of the solid/liquid interface. *Nat. Commun.* 7:12695 doi: 10.1038/ncomms12695 (2016).

## Supplementary Material

Supplementary Information Supplementary Figures 1-15, Supplementary Table 1, Supplementary Notes 1-3, Supplementary References

Peer Review
