# Supplementary material for: Unravelling the electrochemical double layer by direct probing of the solid/liquid interface
Source: Nat Commun. 2016 Aug 31;7:12695. doi: 10.1038/ncomms12695 (PMC5013669; doi:10.1038/ncomms12695)
Supplement: Supplementary Information — Supplementary Figures 1-15, Supplementary Table 1, Supplementary Notes 1-3, Supplementary References [file ncomms12695-s1.pdf]

Supplementary Information

Supplementary Figures

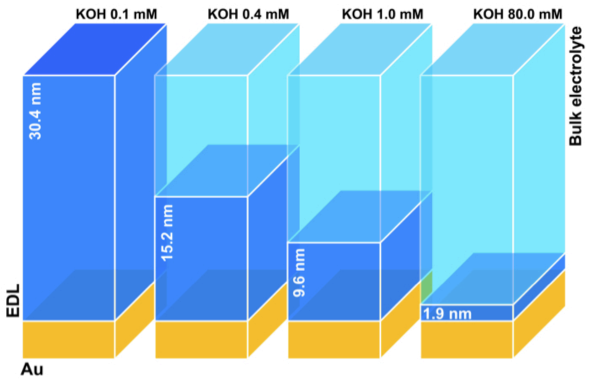

Supplementary Figure 1. Schematic representation of the double layer thickness as a function of the supporting electrolyte concentration, for a total liquid layer thickness at the gold/liquid interface of about 30 nm (EDL: electrochemical double layer).

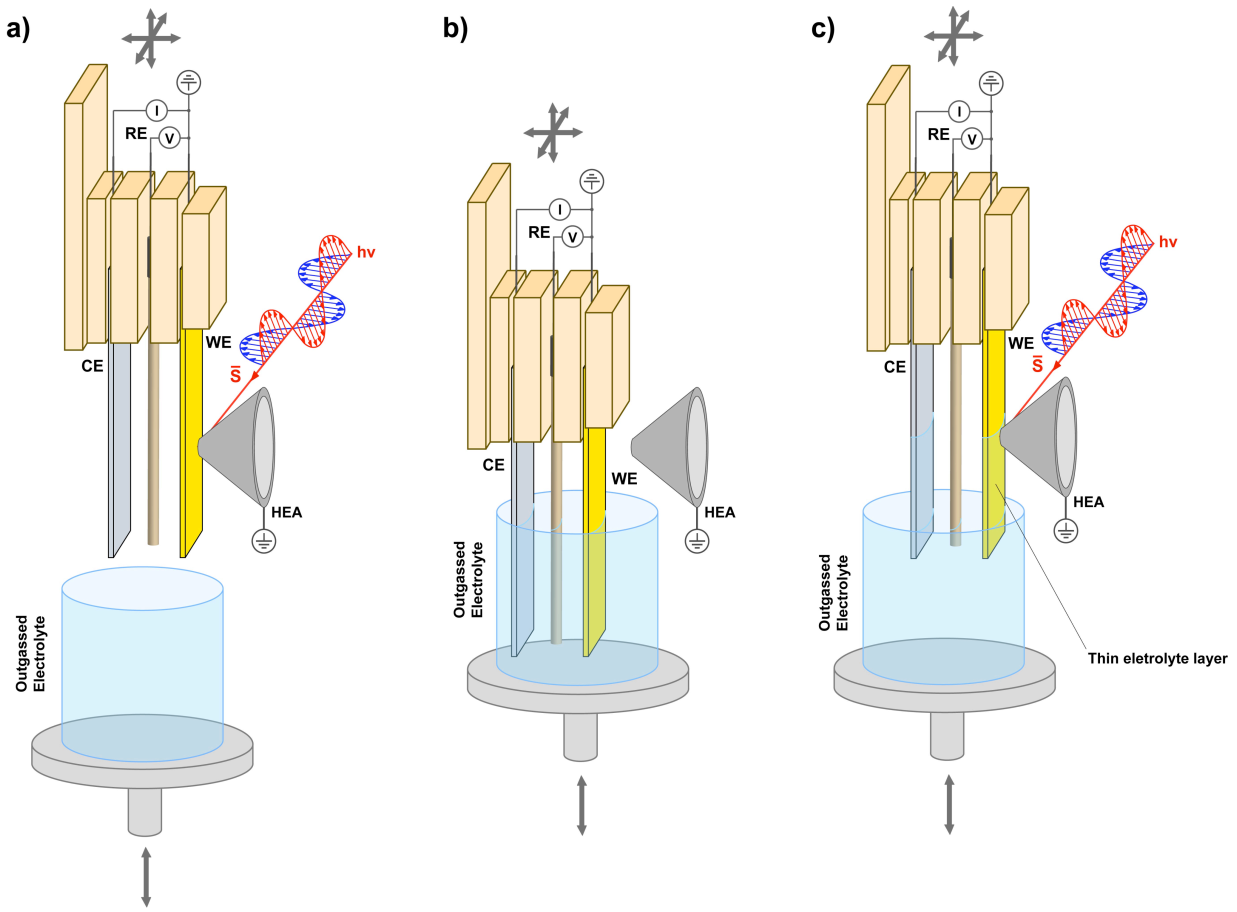

Supplementary Figure 2. Scheme of three-electrode electrochemistry setup in the APXPS chamber available in BL 9.3.1 (WE: polycrystalline Au, CE: polycrystalline Pt, RE: miniaturized Ag/AgCl/$Cl^-_{(sat.)}$); **a**: position of the electrodes before immersion (hydrated conditions); **b**: the electrodes are immersed into the electrolyte (0.1, 0.4, 1.0 and 80.0 mM KOH solution), where any electrochemical treatment can be performed within the APXPS chamber; **c**: after the 'dip and pull' procedure, the electrodes are finally brought to the *operando* APXPS position (chronoamperometry is performed at different potentials within the double layer region, while N 1s , O 1s and Au 4f core levels are measured) (APXPS: ambient pressure X-ray photoelectron spectroscopy; HEA: hemispherical electron analyzer).

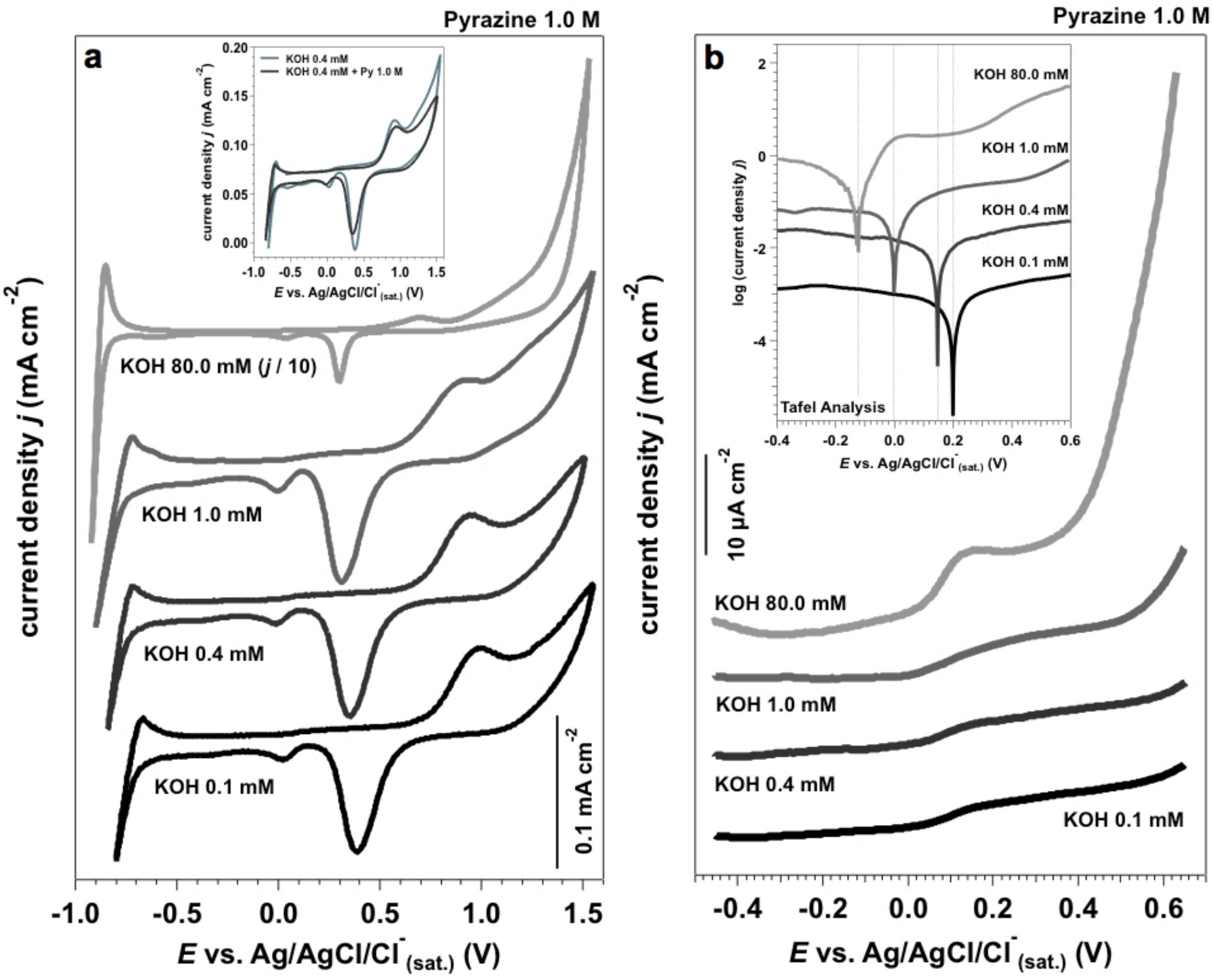

Supplementary Figure 3. a: Cyclic voltammetry (CV) results obtained for gold WE in different KOH concentrations (v = 20 mV/s). The inset shows that the addition of 1.0 M pyrazine does not influence the double layer potential region; **b**: linear sweep voltammetries (LSV) over the double layer region (v = 5 mV/s). The inset reports the corresponding Tafel analysis.

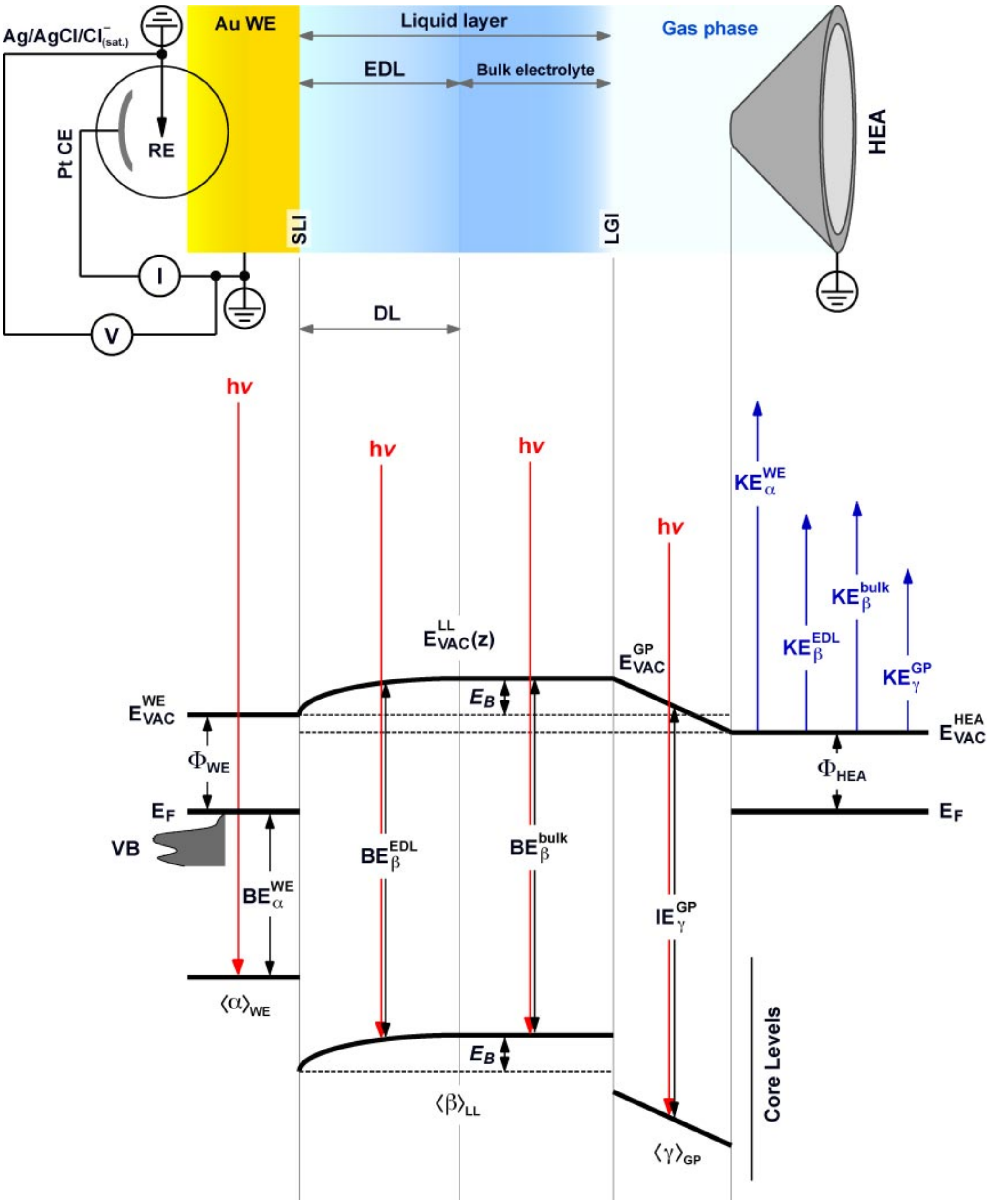

Supplementary Figure 4. Energy diagram at the solid-liquid (SLI) and liquid-gas interface (LGI), as studied during the *operando ambient pressure* X-ray photoelectron spectroscopy measurements (WE: working electrode; RE: reference electrode; CE: counter electrode; HEA: hemispherical electron analyzer; VB: valence band; BE: binding energy; KE: kinetic energy; IE: ionization energy; LL: liquid layer; GP: gas phase; E_B: nominal applied potential (at the bulk electrolyte); Φ denotes the work function).

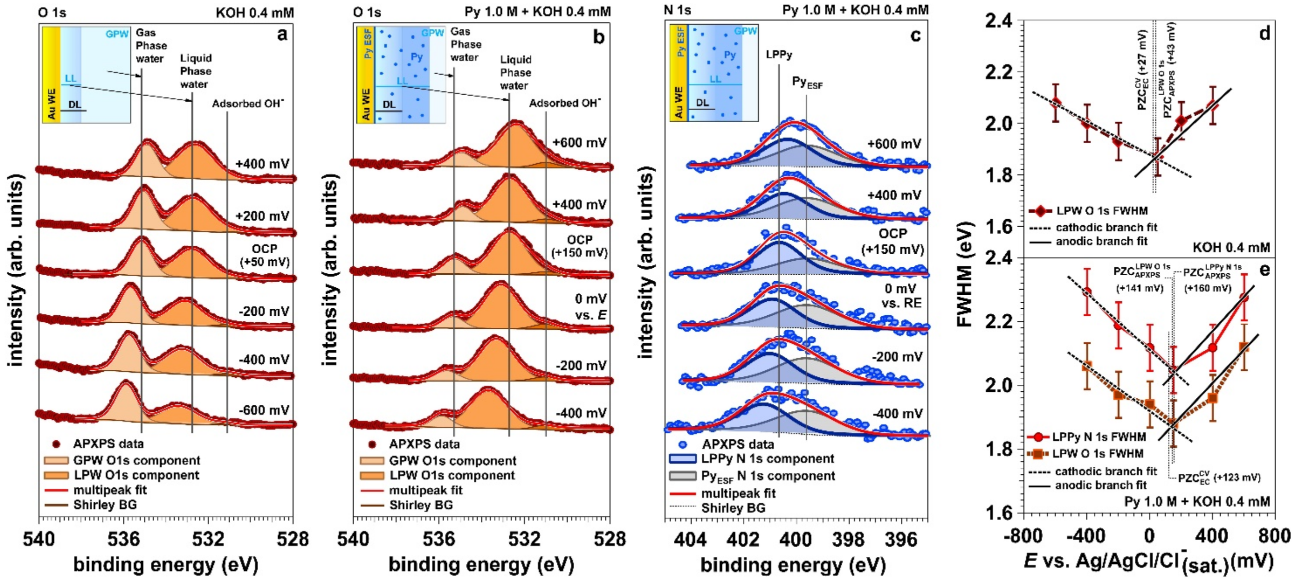

Supplementary Figure 5. Comparison between using water and pyrazine for the electrochemical double layer (EDL) probing; **a, b**: O 1s core level peaks (from water) as a function of the applied potential, for 0.4 mM KOH and 0.4 mM KOH + 1.0 M pyrazine aqueous solutions, respectively; **c**: N 1s core level peaks as a function of the applied potential, for 0.4 mM KOH + 1.0 M pyrazine aqueous solution; **d**: PZC determination using the linewidth trend of the LPW O 1s component as a function of the applied potential, for the case of the pyrazine-free electrolyte; **e**: comparison between the FWHM trends for the LPW O 1s and the LPPy N 1s components (GPW: gas phase water; LPW: liquid phase water; LPPy: liquid phase pyrazine; LL: liquid layer; DL: double layer; OCP: open circuit potential; PZC: potential of zero charge; BG: background; CV: cyclic voltammetry; EC: electrochemistry; APXPS: ambient pressure X-ray photoelectron spectroscopy; FWHM: full-width at half-maximum).

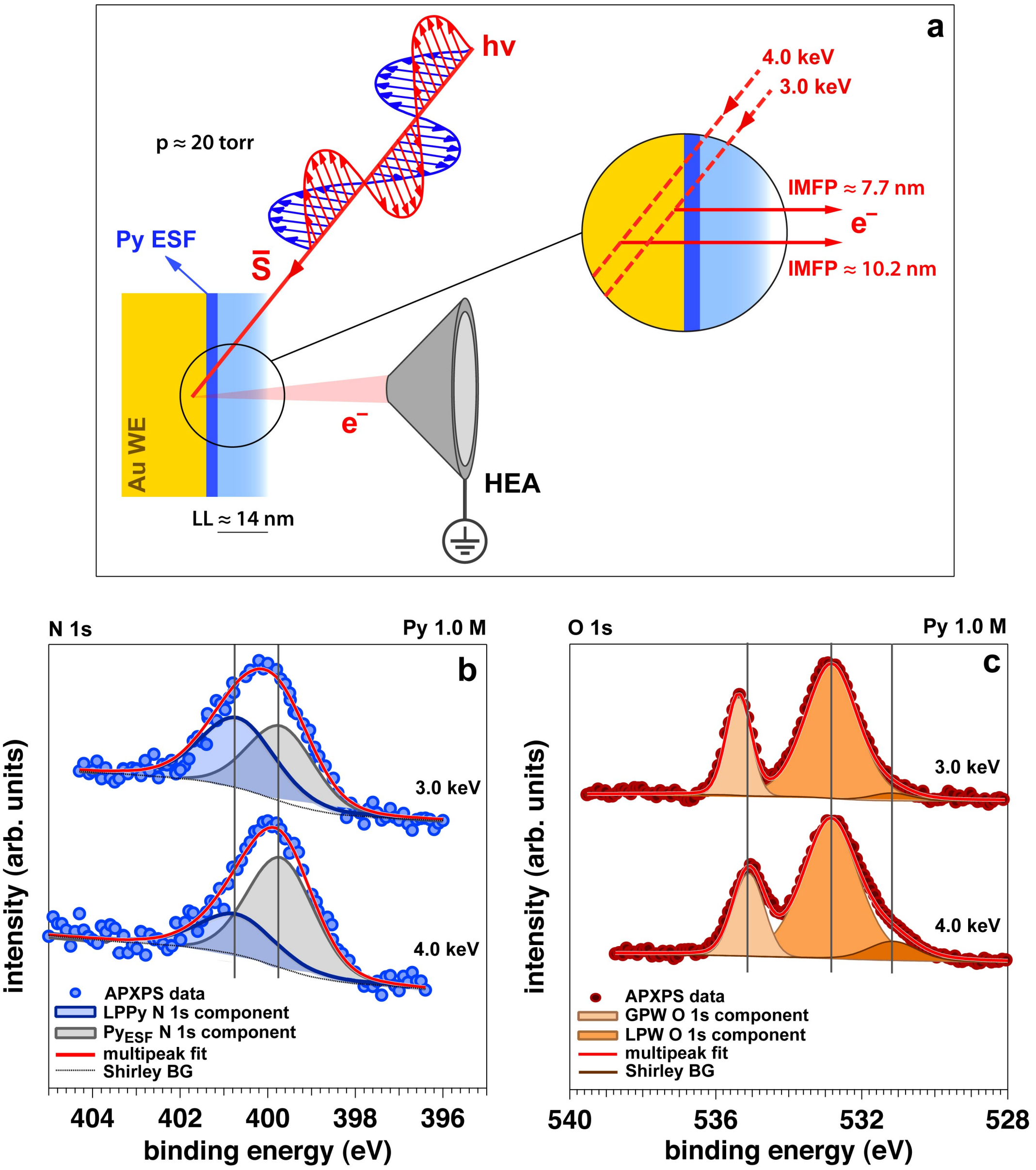

Supplementary Figure 6. Characterization of the Py_{ESF} (pyrazine at the electrode surface) by changing the energy of the incoming photon; **a**: scheme reassuming the different inelastic mean free paths (IMFP) reached for different excitation energies; **b**, **c**: N 1s and O 1s core level peaks acquired at 3.0 and 4.0 keV (GPW: gas phase water; LL: liquid layer; LPW: liquid phase water; LPPy: liquid phase pyrazine; WE: working electrode; BG: background; HEA: hemispherical electron analyzer).

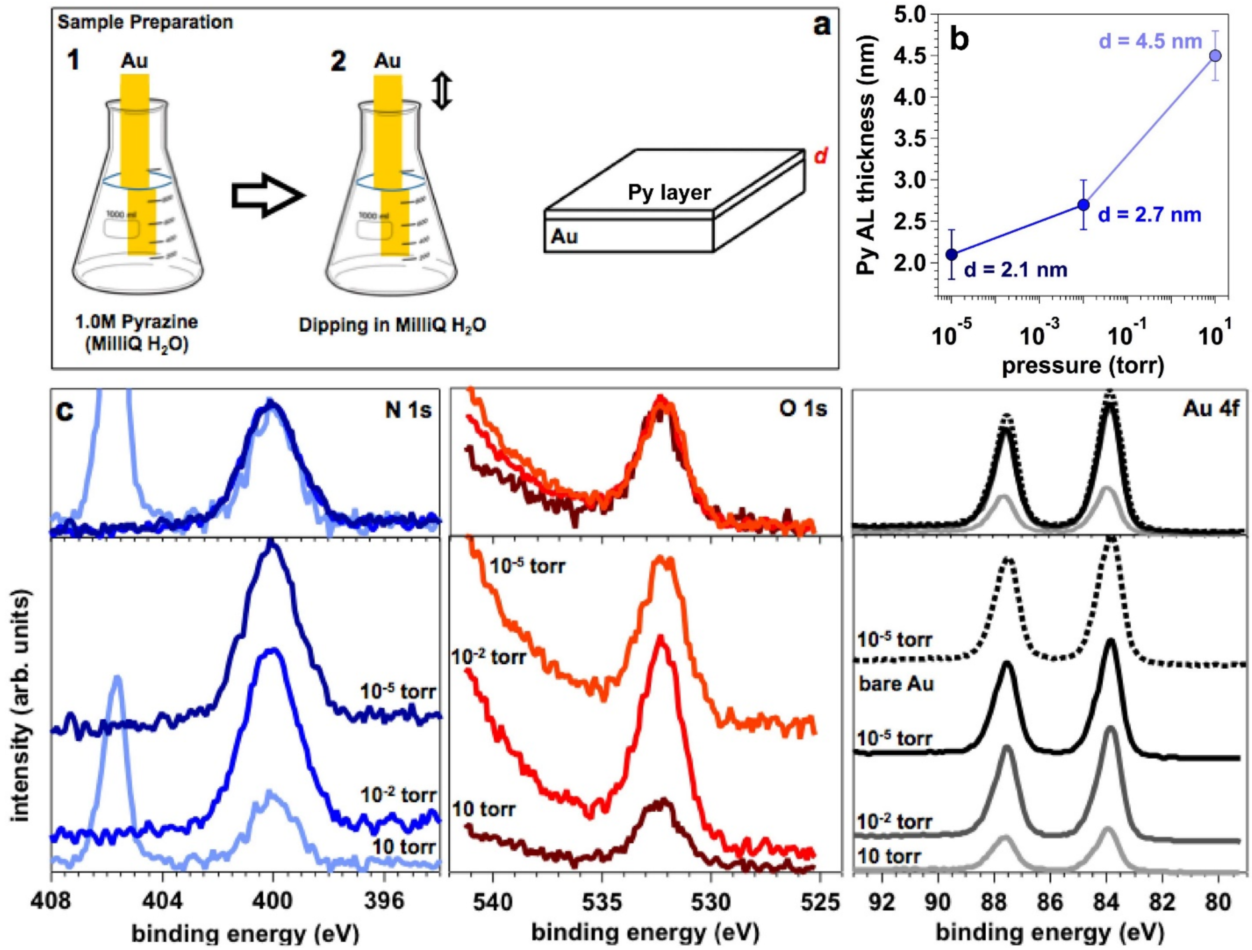

Supplementary Figure 7. a: preparation of the Py layer at the WE surface, by dipping a clean Au sample in a 1.0 M pyrazine aqueous solution; **b**: evaluation of the layer thickness as a function of the pressure; **c**: XPS results for N 1s, O 1s and Au 4f photoelectron peaks, at different working pressures. The attenuation of the Au 4f photoelectron peak was used for the determination of the layer thickness (Py: pyrazine; Py AL: pyrazine adsorbed layer).

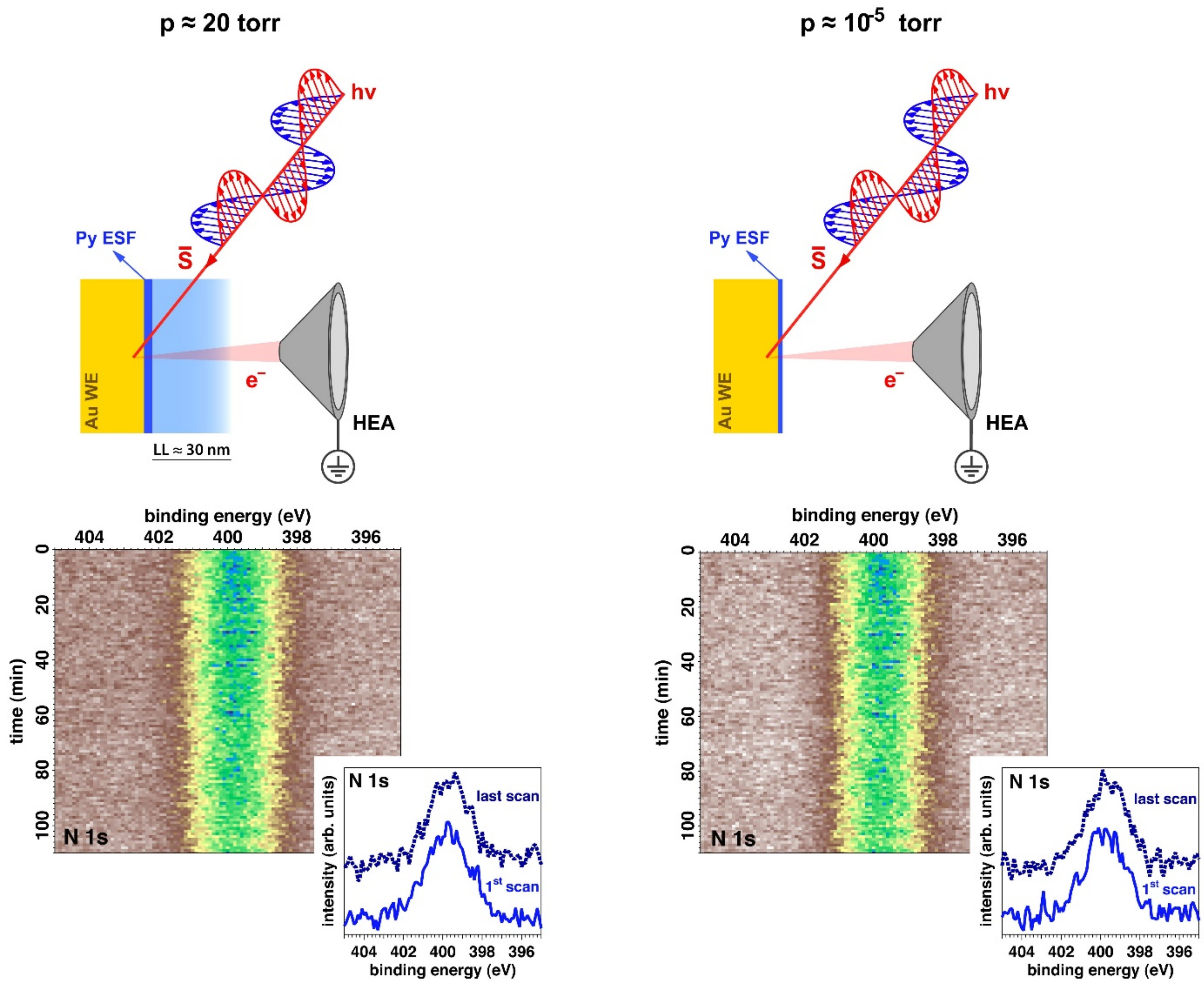

Supplementary Figure 8. Stability measurements of the pyrazine layer at electrode surface (Py_{ESF}) in liquid phase (0.4 mM KOH solution, p ≈ 20 torr) and in high vacuum (p ≈ 10^{-5} torr) (LL: liquid layer; WE: working electrode; HEA: hemispherical electron analyzer).

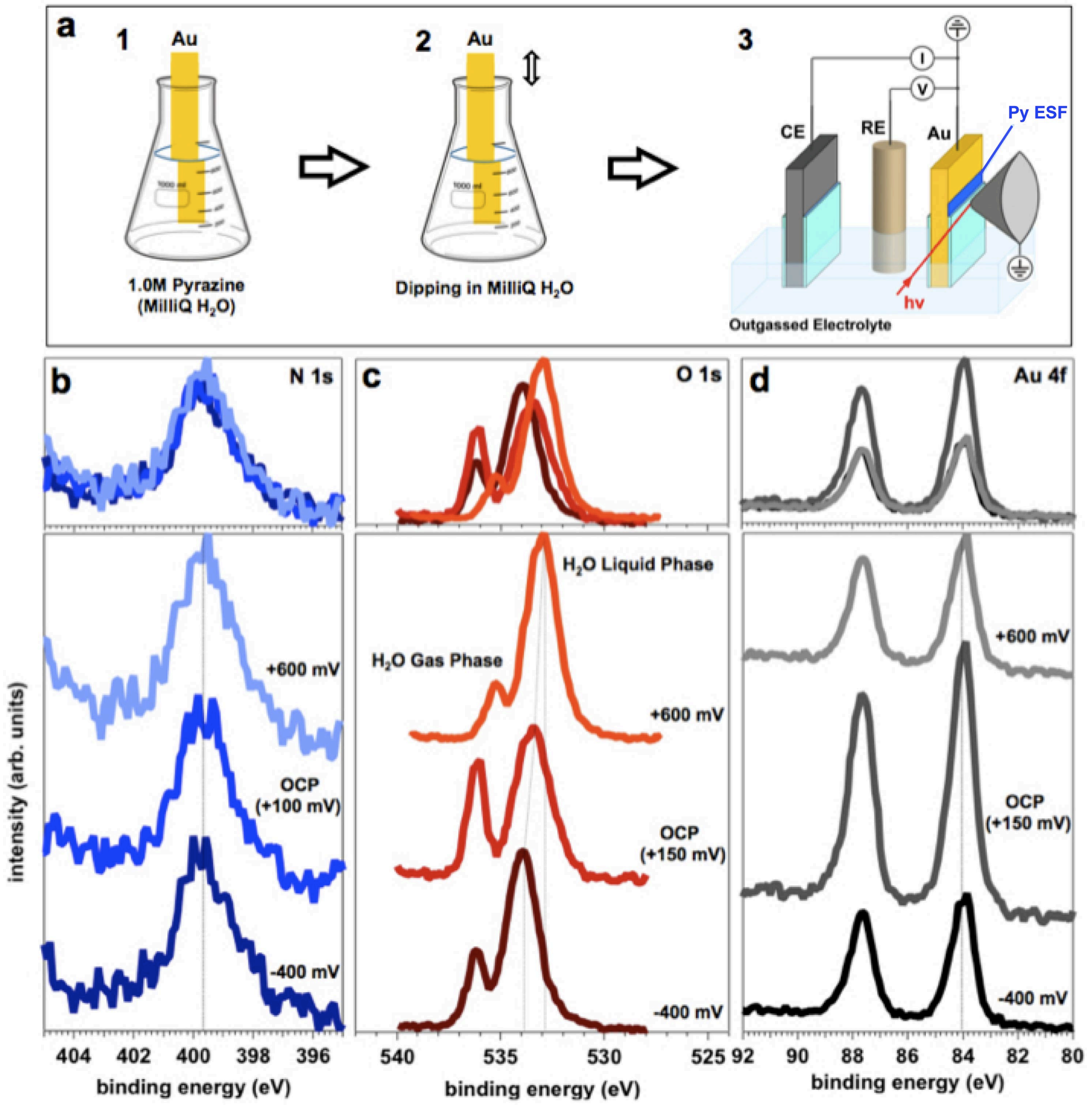

Supplementary Figure 9. Polarization measurements of the pyrazine layer at electrode surface (Py_{ESF}) on the gold working electrode (WE); **a:** schematic representation of the procedure: electrochemical *operando* ambient pressure X-ray photoelectron spectroscopy (APXPS) was performed immersing the previously prepared Au WE/Py_{ESF} system into a pyrazine-free 0.4 mM KOH aqueous solution; **b, c, d:** APXPS results for N 1s, O 1s and Au 4f photoelectron peaks, respectively (CE: counter electrode; RE: reference electrode; OCP: open circuit potential).

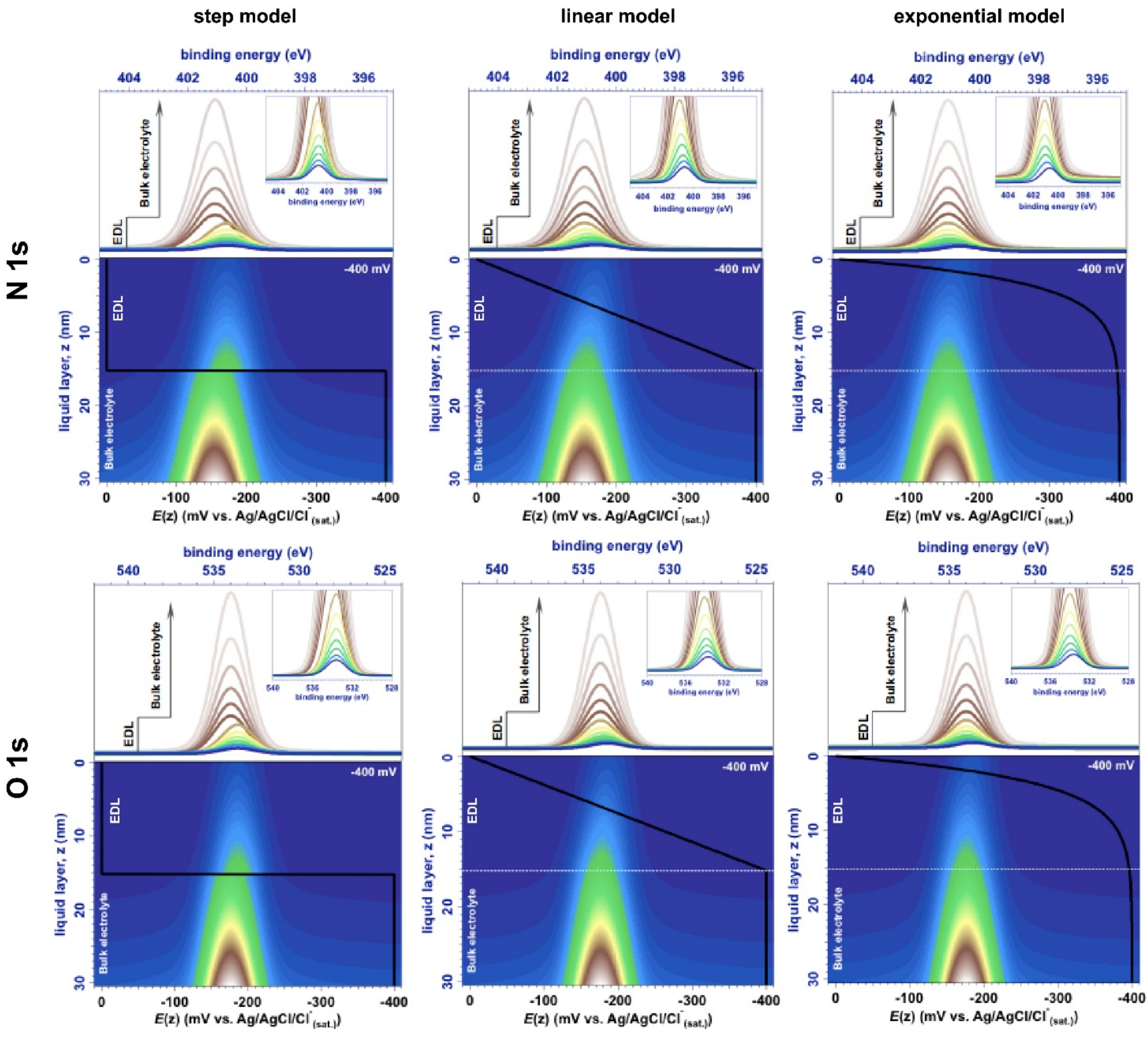

Supplementary Figure 10. Numerical simulations of the potential drop within the electrochemical double layer (EDL) for an applied potential of -400 mV and a KOH concentration of 0.4 mM, corresponding to an EDL thickness (d_{EDL}) equal to 15.2 nm (the total thickness of the liquid layer was set equal to 30 nm, see section S5). The simulations were generated using the three different models of the EDL potential drop, based on a step, linear and exponential functional dependency. The top and bottom part reports the result obtained for the N 1s and the O 1s photoelectron peaks, respectively.

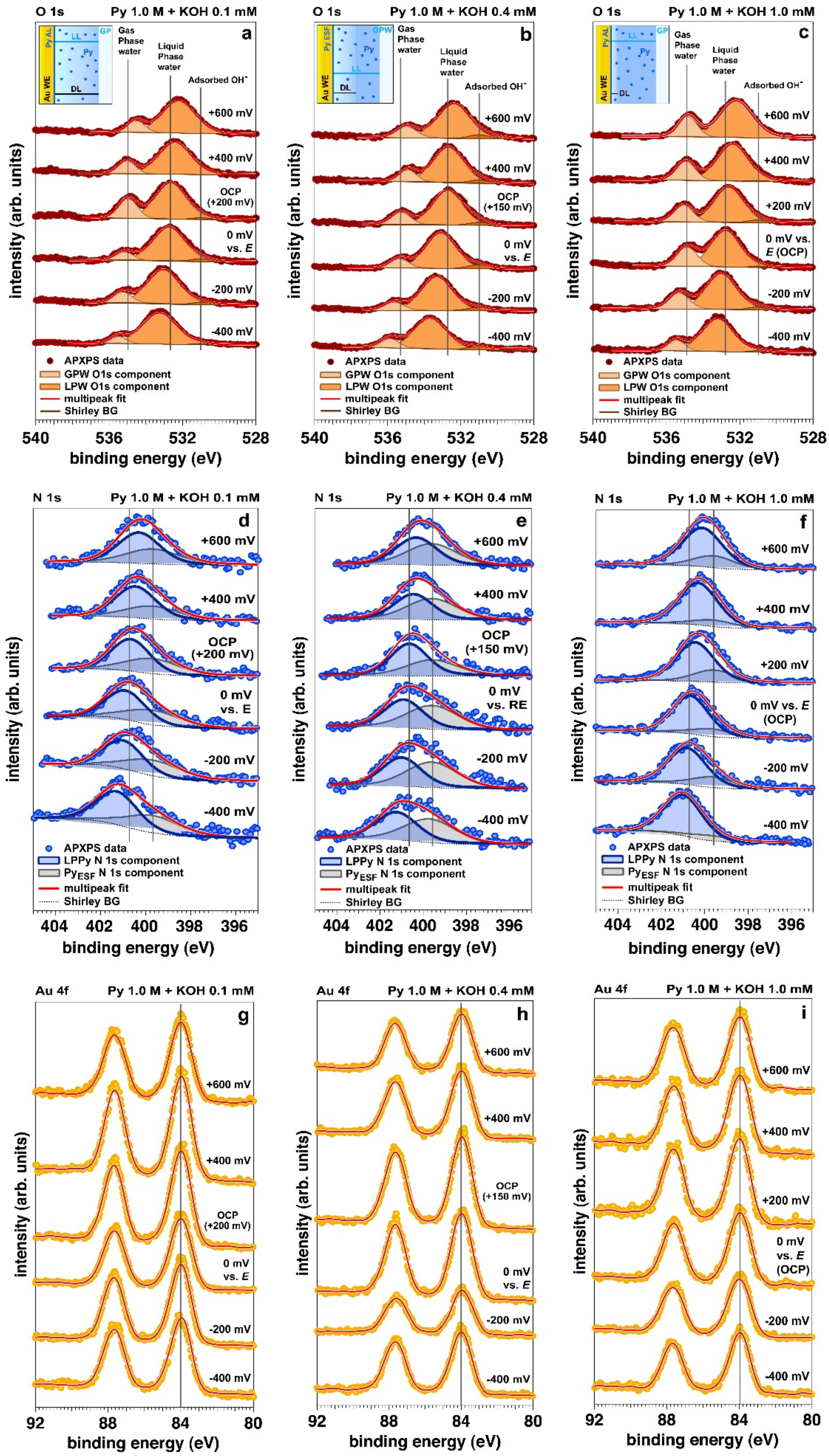

Supplementary Figure 11. Results obtained for the O 1s (**a, b, c**), N 1s **(d, e, f)** and Au 4f (**g, h, i**) photoelectron peaks, as function of the applied potential and for a KOH concentration equal to 0.1 mM (**a, d, g**), 0.4 mM (**b, e, h**) and 1.0 mM (**c, f, i**). Under these conditions, the thickness of the EDL is equal to 30.4 nm, 15.2 nm and 9.6 nm, respectively (Py: pyrazine; Py_{ESF}: pyrazine at the electrode surface; DL: double layer; GPW: gas phase water; LL: liquid layer; LPW: liquid phase water; LPPy: liquid phase pyrazine; WE: working electrode; OCP: open circuit potential; BG: background).

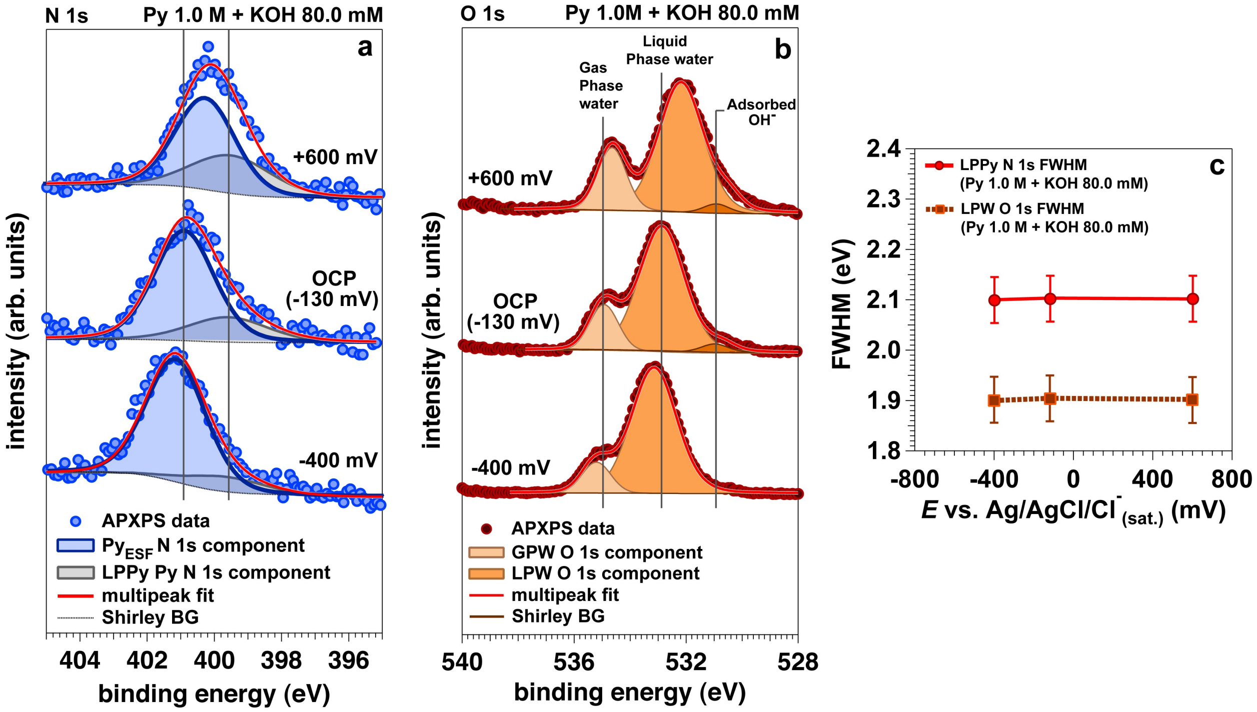

Supplementary Figure 12. Comparison between N (**a**) and O 1s (**b**) photoelectron peaks at different applied potentials for a KOH concentration of 80.0 mM (EDL thickness equal to 1.9 nm). Figure **c** well documents the absence of FWHM modulation for an EDL thickness of 1.9 nm (GPW: gas phase water; LPW: liquid phase water; LPPy: liquid phase pyrazine; LL: liquid layer; DL: double layer; OCP: open circuit potential; BG: background; FWHM: full-width at half-maximum).

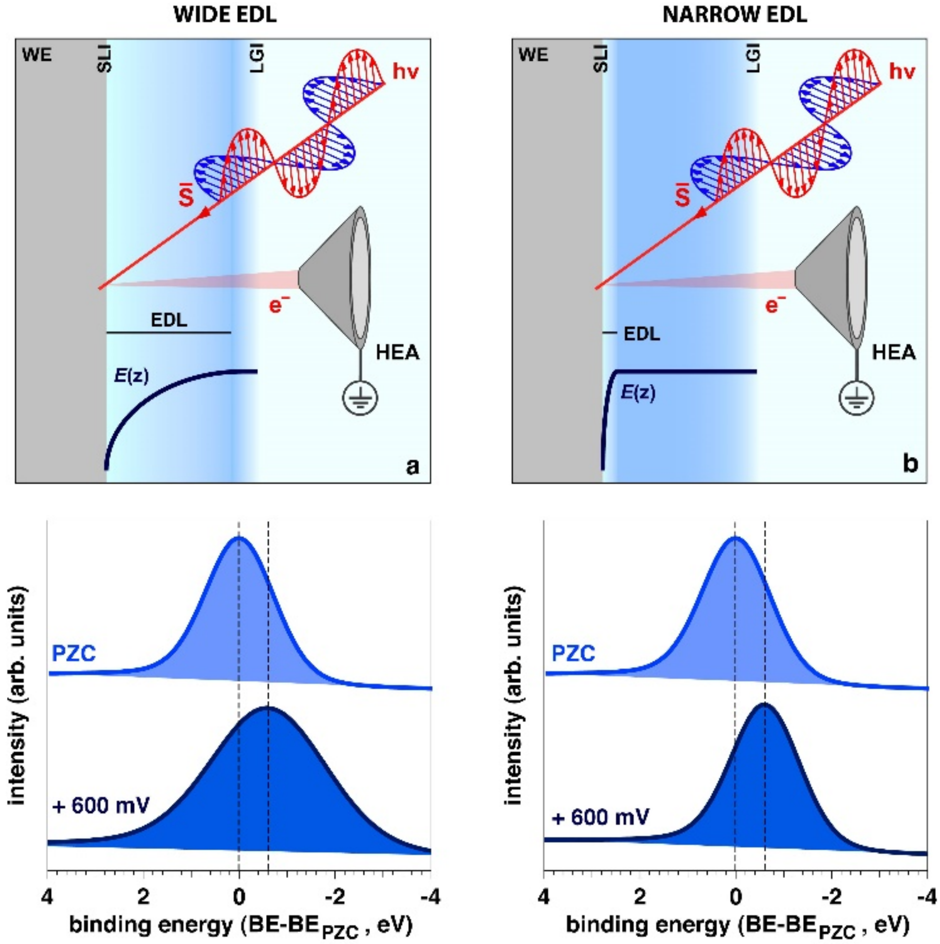

Supplementary Figure 13. Schematization of the observed spectral shift and broadening of the core levels of the elements belonging to the liquid phase, for a wide and narrow EDL (SLI: solid/liquid interface; LGI: liquid/gas interface; PZC: potential of zero charge; BE: binding energy; EDL: electrochemical double layer; WE: working electrode; HEA: hemispherical electron analyzer).

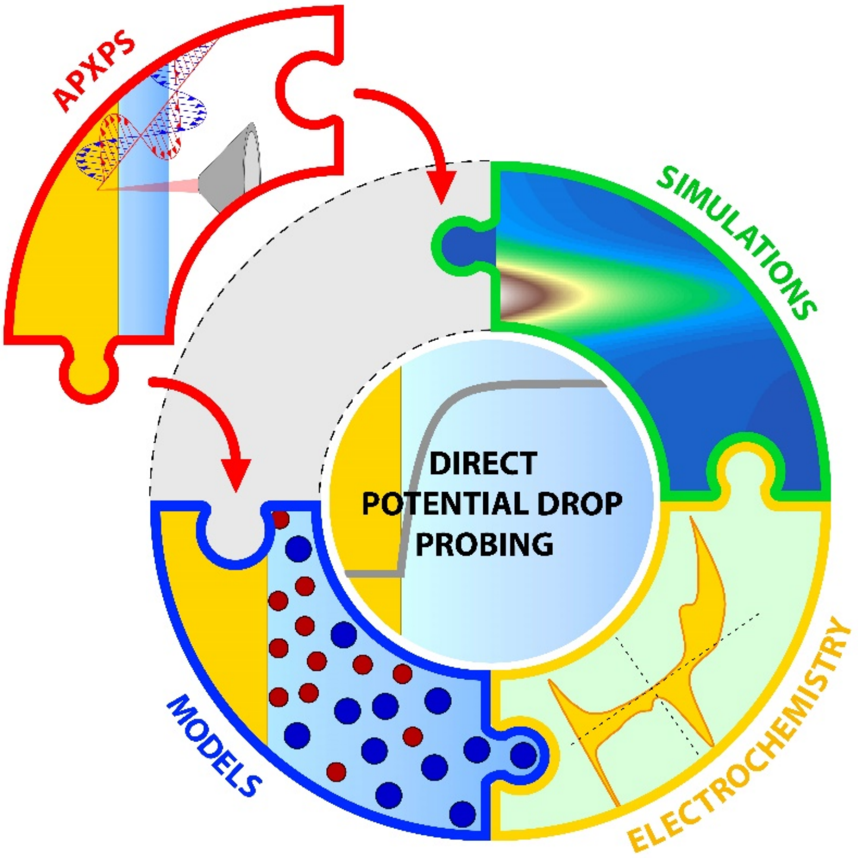

Supplementary Figure 14. Schematization of the combined *operando* APXPS, electrochemical and numerical simulation-based approach for the direct probing of the potential drop within the EDL.

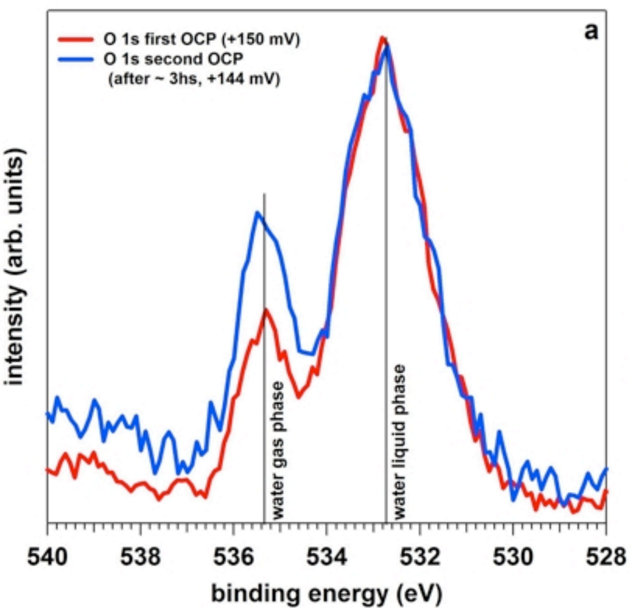

Supplementary Figure 15. O 1s (**a**) and N 1s (**b**) core levels acquired at the OCP with a delay of about 3 hours (i.e. at the beginning and at the end of an experiment where the EDL was probed as a function of the applied potential in an aqueous KOH 0.4 mM solution containing 1.0 M pyrazine). No BE shift over time is detected, as well as shift in the open circuit potential (OCP) (within the electrochemical experimental resolution, about 10 mV).

Supplementary Table

Supplementary Table 1. Chi-square values determined from the difference between the simulated and experimental data, for different electrolyte concentrations (i.e. EDL thicknesses) and using both LPPy N1s and LPW O1s core levels.

	Step function		Linear function		Exponential function	
	**LPPy N1s**	**LPW O1s**	**LPPy N1s**	**LPW O1s**	**LPPy N1s**	**LPW O1s**
**0.1 mM**	9.63	9.02	3.91	4.23	1.89	2.64
**0.4 mM**	7.37	4.89	3.14	3.34	2.13	2.73
**1.0 mM**	8.32	7.76	3.64	3.46	1.98	2.66

Supplementary Notes

Supplementary Note 1.
Characterization of the pyrazine at the electrode surface (Py_{ESF}).

Py adsorption at the electrode/electrolyte interface takes place as a consequence of dipping a clean Au electrode in a Py-containing electrolyte. A pyrazine layer (at the electrode surface, Py_{ESF}) was then obtained on a clean Au polycrystalline surface by dipping the WE in a 1.0 M pyrazine aqueous solution. The system was then studied using different excitation energies, which determines different electron inelastic mean free path (IMFP) as schematically reported in **Supplementary Figure 6 a**. The corresponding N 1s and O 1s core level peaks (for a liquid layer of about 14 nm) are reported in **Supplementary Figure 6 b** and **c**, respectively. From the analysis of the N 1s peak it is possible to observe that the intensity of the Py_{ESF} component undergoes a significant decrease when the photon energy (also known as excitation energy) passes from 4.0 to 3.0 keV, meaning that the former represents indeed a Py-based structure localized at the solid/liquid interface. The Py_{ESF}/LPPy component ratio well documents the described structure, passing from 2.7 to 1.05 as the excitation energy varies between 4.0 and 3.0 keV, respectively. The IMFP at 3.0 keV is almost 25% lower than that achievable using a photon energy of 4.0 keV. Then, the spectral contribution from the chemical species localized at the aforementioned interface, buried by the thin electrolyte layer on the WE surface, undergoes an intensity decrease as the used photon energy decreases as well (according to the Beer-Lambert law, **Supplementary Equation 2**). The described phenomenology is accompanied by a ratio between the adsorbed OH^- and the LPW component exhibiting the same behavior observed for the Py_{ESF}/LPPy component ratio. As the excitation energy changes from 4.0 to 3.0 keV, the adsorbed OH^-/LPW component ratio varies in fact from 8.4 to 23.5.

To gain further insights regarding to the Py ESF chemical species, the pyrazine layer was then extensively characterized by means of XPS at different working pressures (from 10 torr of water gas phase down to 10^{-5} torr). Interestingly, as reported in **Supplementary Figure 7 b,** the thickness of the Py layer decreases together with the pressure, most likely due to the water evaporation and an overall adjustment of the adsorption configuration. Moreover, as it is possible to observe from **Supplementary Figure 7 c**, by increasing the vacuum level no changes are observed as regards to the spectral position (about 399.7 eV) and the shape of the N 1s and O 1s core levels. At the same time, an expected loss of liquid phase/adsorbed water takes place (confirmed by the increase of the Au 4f core level as the pressure decreases).

The time stability and the eventual induced radiation damage of the Py layer have been also characterized, as reported in **Supplementary Figure 8**. As it is possible to observe, the Py layer is stable and it does not undergo radiation damage effects both in liquid phase (about 20 torr) and at a pressure of 10^{-5} torr. These findings mean that the bond strength between pyrazine and gold is relatively high, as well reported in literature for Au (6) as well as for semiconductors and oxidic substrates (1, 2).

To rule out possible polarization effects of the Py layer with the applied potential, electrochemical operando APXPS was performed immersing the previously prepared Au WE/Py layer system into a pyrazine-free 0.4 mM KOH aqueous solution, as schematically reported in **Supplementary Figure 9 a**. As it is possible to observe from **Supplementary Figure 9 b**, no shifting or broadening of the N 1s photoelectron peak is detectable with the applied potential (the potential control within the nanometric-thick electrolyte layer on the WE is ensured by the rigid shift of the O 1s photoelectron peak, **Supplementary Figure 9 c**, as the applied bias varies along the double layer region).

Supplementary Note 2.
EDL probing in a pyrazine free- and containing- 0.4 mM KOH electrolyte.

Pyrazine (3, 4) (Py) is particularly well suited to probe the properties of the solid/liquid interface due to the absence of a permanent dipole moment, together with its electrochemical inertness, which does not influence the surface potential or the potential drop within the diffuse layer (5, 6). Moreover, the presence of two N atoms 'labeling' the molecular probe provides a chemically specific element that can be probed with XPS. This choice can become crucial for the interfacial physical chemistry investigation in complex electrochemical environments, such as the solid electrolyte interface (SEI) (7, 8) in batteries or the biological interfaces at the cellular membranes (9, 10). In particular, in a given BE range an overlap of different elements can take place (e.g. B 1s/P 2s, O 1s/V 2p, Mo 3d/S 2s, Zr 4s/Li 1s etc., to list a few examples), leading to a difficult (or unambiguous) discrimination between the pure chemical shift effects and the spectral fine features caused instead by the potential drop within the diffuse layer. Thus, we will demonstrate the use of both pyrazine (N 1s) and a solvent-related core level (in this case O 1s) to probe the EDL property of the solid/liquid junction.

To further understand the EDL and substantiate the viability of a molecular probe, we studied the potential drop within the EDL with and without the molecular probe, as reported in **Supplementary Figure 5 a** and **b-c**, respectively. The O 1s photoelectron spectra were de-convoluted by using three different chemically shifted components attributed to gas phase water (GPW), liquid phase water (LPW) and adsorbed hydroxyls at the solid/liquid interface (from highest to lowest BE, respectively). The BE of the adsorbed hydroxyls is centered at 530.9 eV, and does not shift with the applied potential as similarly observed for the Py at the electrode surface (Py_{ESF}, **Supplementary Figure 5 a** and **b**). It is possible to observe the broadening modulation of the LPW O 1s photoelectron peak as function of the applied potential leads to a V-shape for both Py and Py-free electrolytes (**Supplementary Figure 5 d** and **e**) with the minimum value closely centered at the PZC. Where the Py-free electrolyte electrochemically determined potential of zero charge (PZC) is +27 mV, whereas the PZC determined by the linear interpolation of the FWHM values is +43 mV.

The same phenomenology is also observed for the LPW O 1s photoelectron in case of the pyrazine-containing electrolyte, as shown in **Supplementary Figure 5 e**. The broadening of the LPW component shows the same V-shape behavior as a function of the applied potential, allowing the same PZC determination as performed using the spectral broadening of the liquid phase Py (LPPy) N 1s component (**Supplementary Figure 5 c** and **e**). Additionally, the spectroscopy-based (LPW O1s) and the electrochemically determined PZC values are in good agreement between each other (+141 mV vs. +123 mV, respectively). Moreover, a good agreement exists between the potential of zero charge determined with the molecular probe and the direct use of a solvent-related core level, with a difference between the values of 19 mV (far below the total spectral resolution of 100 mV). This observation leads to the conclusion that our operando electrochemical APXPS allows the direct probing of the electric field within the diffuse layer. In particular, we have demonstrated that information can be extracted by using either a solvent-related core level or a suitably chosen molecular probe.

It is noteworthy considering that the PZC values on gold for the Py-containing and -free electrolytes are slightly different, as clear from their determination both via the CV and MWEM method. This phenomenology can be rationalized by considering that the

presence of Py may weakly influences the chemical potential properties of the WE/electrolyte interface. However, it is important to highlight two things: firstly, that this weak interaction does not influence the potential drop profile nor electrochemical properties, just the PZC and the open circuit potential (OCP) values; secondly, that it is possible to track these weak effects by spectroscopy, paving the way toward the probing of different electrochemical systems for which the PZC can be influenced by strongly adsorbed layer or particular surface orientations/terminations.

Supplementary Note 3.
Au 4f, O 1s and N 1s photoelectron peaks as a function of the applied potential and the electrolyte concentration.

The thickness (d) of the electrolyte layer on the WE (see the schematization reported in **Supplementary Figure 1**) was determined by using the Beer-Lambert law applied to the Au 4f intensity attenuation, as reported by **Supplementary Equation 1**:

$$d = -\Lambda_e \ln\left(\frac{I_{APXPS}}{I_{HC}}\right)$$ **Supplementary Equation 1**

The Λ_e is the IMFP for the escaping photoelectron generated by the photoionization of the Au $4f_{7/2}$ core level, at a BE of 84.0 eV. Considering an incoming photon energy of 4.0 keV and a 1.0 M pyrazine aqueous solution as attenuating medium, the Tanuma-Powell-Penn algorithm gives a Λ_e of 10.2 nm. I_{HC} and I_{APXPS} are the Au 4f integrated photoelectron intensities for the Au sample exposed to water vapor (hydrated conditions, HC) and under APXPS conditions (after the dip and pull procedure), respectively. The dependency from the take-off angle is unitary, since the experiments were conducted under NE acquisition conditions.

The overall results obtained by varying the applied potential across the double layer region for different KOH concentrations (0.1 mM, 0.4 mM and 1.0 mM, which determine a EDL thickness (d_{EDL}) of 30.4 nm, 15.2 nm and 9.6 nm, respectively) are reported in **Supplementary Figure 11**. For the evaluation of the film thickness, we have used **Supplementary Equation 1** and the integrated areas corresponding to the Au 4f photoelectron peaks reported below; the electrolyte layer mean thickness, by averaging the d values over the different applied potential, is equal to 30 ± 2 nm.

For high KOH concentrations (d_{EDL} < 2 nm), the contribution from the molecules within the bulk electrolyte dominates in the N 1s spectral convolution, leading to a rigid spectral shift and to a weaker broadening of the photoelectron peaks (as function of the applied potential) the higher the KOH concentration (in line with our previous studies performed at higher electrolyte concentrations). **Supplementary Figure 12** well documents this phenomenology: for a KOH concentration of 80 mM (d_{EDL} = 1.9 nm), it is possible to observe a rigid shift of the N (**Supplementary Figure 12 a**) and O 1s (**Supplementary Figure 12 b**) photoelectron peaks as function of the applied potential to the WE. **Supplementary Figure 12 c** shows in fact no FWHM modulation (within the experimental error) as function of the applied potential.

Supplementary References

1. Lee, H.-K., Park, J., Kim, I., Kim, H.-D., Park, B.-G-, Shin, H. J. *et al.* Selective reactions and adsorption structure of pyrazine on Si(100): HRPES and NEXAFS study. *J. Phys. Chem. C* **116,** 722–725 (2012).

2. Omiya, T., Yokohara, H. & Shimomura, M. Well-oriented pyrazine lines and arrays on Si(001) formed by thermal activation of substrate. *J. Phys. Chem. C* **116,** 9980–9984 (2012).

3. Wheatley, P. J. The crystal and molecular structure of pyrazine. *Acta Cryst.* **19,** 182–187 (1957).

4. Billes, F. & Mikosch, H. Protonation and solvation of pyrazine. *Structural Chemistry* **5**, 307–319 (1992).

5. Conway, B. E., Angerstein-Kozlowska H. & Dhar, H. P. On selection of standard states in adsorption isotherms. *Electrochim. Acta* **19,** 455–460 (1974).

6. Iannelli, A., Richer, J. & Lipkowski, J. Adsorption of pyrazine at the polycrystalline gold-solution interface. *Langmuir* **5,** 466–473 (1989).

7. Goodenough, J. B. & Kim, Y. Challenges for rechargeable Li batteries. *Chem. Mater.* **22,** 587–603 (2010).

8. Jarry, A., Gottis, S., Yu, Y.-S., Roque-Rosell, J., Kim, C., Cabana, J. *et al.* The formation mechanism of fluorescent metal complexes at the $Li_xNi_{0.5}Mn_{1.5}O_{4-\delta}$/carbonate ester electrolyte interface. *J. Am. Chem. Soc.* **137,** 3533–3539 (2015).

9. Krysinski, P. & Ti Tien, H. Membrane electrochemistry. *Membrane Sci. Tech.* **5,** 283–348 (2000).

10. Moulton, S. E., Barisci, J. N., Bath, A., Stella, R. & Wallace, G. G. Studies of double layer capacitance and electron transfer at a gold electrode exposed to protein solutions. *Electrochem. Acta* **49,** 4223–4230 (2004).
