# Supplementary material for: Unravelling the electrochemical double layer by direct probing of the solid/liquid interface
Source: Nat Commun. 2016 Aug 31;7:12695. doi: 10.1038/ncomms12695 (PMC5013669; doi:10.1038/ncomms12695)
Supplement: Peer Review [file ncomms12695-s2.pdf]

Reviewers' Comments:

Reviewer #1 (Remarks to the Author)

The manuscript reports on the direct measurement of solid/liquid electrochemical double layer (EDL) by using operando tender x-ray ambient pressure XPS. By comparing FWHM information of AP-XPS results with simulation, the potential drop within of solid/liquid EDL is directly probed quantitatively.
The manuscript is carefully written with clear expressions and through experimental analysis. (I found the SI is a bit too long, but it is necessary.)

I find their findings are novel enough to warrant the publication in Nature Comm. A few novel aspects of their approach and findings are following.

1. To probe the solid/liquid interfacial properties, the group used the electrochemically inert and non-polar Pyrazine, which I found the key to the success of this experiment and a brilliant choice.
2. The control/maintenance of nanometric thin liquid films is critical to the success of experiment and the group clearly demonstrates the stability of thin liquid layer during the measurement.
3. This is the very first EDL measurement of liquid/solid interface under the operating condition, and the various scientific communities, especially electrochemistry group, will gain much benefits from this approaches.

One aspect that I would like to comment is on the application of SW-AP-XPS. In the text, SW-AP-XPS is recommended to get the higher spatial resolution near electrode surface. From my back-of-envelope calculation, the estimated Bragg angle, based on the combination of standing wave mirror with the periodicity of 3nm and 4000 eV photon energy, becomes ~2.8 degree from the electrode surface, which can be achieved with high-precision manipulator. However, the analysis of FWHM applied in this manuscript may not longer valid since electric fields from standing wave will be modulated inside of EDL region.

Additionally, M. Brown et al. previously reported on the EDL structures of electrolyte/nanoparticles interfaces utilizing liquid-jet AP-XPS, e.g. Phys. Rev. X, 2016, 6, 011007, Angew. Chem. Int. Ed. 2016, 55, 3790. However, the experimental approaches and analysis of those reports are very different from this manuscript and does not contain the information on EDL of lquid/solid interface.

Since the manuscript covers all the questions that I had, I don't have any comments for further correction or addition.

Reviewer #2 (Remarks to the Author)

Summary of the key results
In the manuscript "Unraveling Electrochemical Double Layer by Direct Probing of the Solid/Liquid Interface" Favaro et al present an experimental study of the electrochemical double layer (EDL) using ambient pressure X-ray photoelectron spectroscopy (APXPS) and the "dip'n'pull" method. The key result is that the broadening of the core-lines to exhibit a broadening when a voltage is applied to the working electrode, which is interpreted as a manifestation of the potential drop over the EDL. From the dependence of the broadening as function of applied voltage, the potential of zero charge (PZC) was determined, and found to be in good agreement with values from conventional electrochemical characterization.

Data & methodology:
The authors use an advanced and relatively new method, which is capable of providing detailed microscopic information. The approach is, as far as I can tell, valid, and the quality of data and presentation are very good. I do not, however, agree to the authors use of the term "operando". My understanding is that this is used within the community to describe conditions in which a device is operating, i.e. performing some function, e.g. a catalytic reaction. As far as I see, the present measurements should rather be described as "in situ".
The authors claim to have developed the "minimum width extrapolation method" (MWEM). To me it appears to consist of fitting two straight lines to the two halves of the data set. If there was any other development, this should be explained.
In figure 4, the third row is said to report "the exponential potential drop within the EDL as function of both the concentration and the applied potential, directly derived from the comparison between the experimental data and the numerical simulations". How this derivation was made is unclear, and needs to be explained better.

Appropriate use of statistics and treatment of uncertainties
The discussion on different models for the EDL, as presented in fig 3, concludes that an exponential model describes the data best. This may well be true, but considering the error bars, at least the linear model cannot be excluded.

Conclusions:
The main conclusion, i.e. that the potential drop over the EDL can be probed with APXPS, seems robust and valid.
Clarity and context: The manuscript is well written, and the reasoning is easy to follow. The problem and the result are put into a larger context in a good way.

Reviewer #3 (Remarks to the Author)

The manuscript by Favaro et al. describes probing the electrochemical solid/liquid interface and the electrochemical double-layer (DL) both by electrochemical methods and by operando ambient pressure XPS (APXPS). This manuscript shows excellent new and original results, clearly demonstrating that the DL and the potential drop within the DL can be probed spectroscopically under operando electrochemical conditions overcoming many issues related to previous studies, where mainly the DL was simulated ex-situ in a UHV environment. I enjoyed reading this manuscript and there are not too many comments to be given. This is a very important work pathing the way to a significant better understanding of the DL and all the processes occurring at the solid-liquid interface...and I am surprised that this work is not published in one of the higher impact journals of the Nature family.
1. The main question related to this work relates to one important point: Is a charging of the electrolyte observed during the XPS measurements as e.g., observed within ionic liquids (see e.g., ECS Electrochemistry Letters 2(4)(2013) H13-H15) and how was it dealt with? Could charging effects also lead to distortions of the potential distribution within the DL resulting from core level shifts? As the ms is written, it appears this is not the case, but this important point needs to be clarified and the authors need to explain, why this may not be the case.
2. Besides the summary of the previous work on DLs, the authors should consider to also discuss specifically the older ex-situ UHV approaches and outline what additional information can be drawn from the operando XPS work (e.g., the work by Wagner & Ross, but also other summarized in ref. 18 of this ms.)
3. This is mainly a comment towards the format: Even in the Letter size printout, figures 1, 2 and 3 are very difficult to read as everything is very small and barely visible (e.g., legends and descriptions). Figure 1 needs to be over both columns at least, otherwise information cannot be read.

Reviewers' comments

Reviewer #1 (Remarks to the Author)

The manuscript reports on the direct measurement of solid/liquid electrochemical double layer (EDL) by using operando tender x-ray ambient pressure XPS. By comparing FWHM information of AP-XPS results with simulation, the potential drop within of solid/liquid EDL is directly probed quantitatively.

The manuscript is carefully written with clear expressions and through experimental analysis. (I found the SI is a bit too long, but it is necessary.)

I find their findings are novel enough to warrant the publication in Nature Comm. A few novel aspects of their approach and findings are following.

1. To probe the solid/liquid interfacial properties, the group used the electrochemically inert and non-polar Pyrazine, which I found the key to the success of this experiment and a brilliant choice.
2. The control/maintenance of nanometric thin liquid films is critical to the success of experiment and the group clearly demonstrates the stability of thin liquid layer during the measurement.
3. This is the very first EDL measurement of liquid/solid interface under the operating condition, and the various scientific communities, especially electrochemistry group, will gain much benefits from this approaches.

Reply to the referee:
We are grateful to the referee for their appreciation of this work.

One aspect that I would like to comment is on the application of SW-AP-XPS. In the text, SW-AP-XPS is recommended to get the higher spatial resolution near electrode surface. From my back-of-envelope calculation, the estimated Bragg angle, based on the combination of standing wave mirror with the periodicity of 3nm and 4000 eV photon energy, becomes ~2.8 degree from the electrode surface, which can be achieved with high-precision manipulator. However, the analysis of FWHM applied in this manuscript may be no longer valid since electric fields from standing wave will be modulated inside of EDL region.

Reply to the referee:
We appreciate the comments and suggestions from the referee on how to best utilize SW-APXPS in future experiments to probe regions of the EDL closer to the electrode surface (i.e. at the inner/outer Helmholtz layers (IHP-OHP)). Based on these suggestions, we will use the higher

spatial (depth) resolution offered by SW-APXPS to probe specific adsorptions of ions and charge transfers between the electrified WE surface and molecules in solution at the IHP-OHP. We have updated the following text to better delineate future directions:

"…will be explored, in particular toward the study of surface adsorbates, ionic specific adsorptions and charge transfers at the IHP, and their interplay in the faradaic processes occurring at the electrified interface".

Additionally, M. Brown et al. previously reported on the EDL structures of electrolyte/nanoparticles interfaces utilizing liquid-jet AP-XPS, e.g. Phys. Rev. X, 2016, 6, 011007, Angew. Chem. Int. Ed. 2016, 55, 3790. However, the experimental approaches and analysis of those reports are very different from this manuscript and does not contain the information on EDL of liquid/solid interface.

Since the manuscript covers all the questions that I had, I don't have any comments for further correction or addition.

Reviewer #2 (Remarks to the Author):

Summary of the key results:
In the manuscript "Unraveling Electrochemical Double Layer by Direct Probing of the Solid/Liquid Interface" Favaro et al present an experimental study of the electrochemical double layer (EDL) using ambient pressure X-ray photoelectron spectroscopy (APXPS) and the "dip and pull" method. The key result is that the broadening of the core-lines to exhibit a broadening when a voltage is applied to the working electrode, which is interpreted as a manifestation of the potential drop over the EDL. From the dependence of the broadening as function of applied voltage, the potential of zero charge (PZC) was determined, and found to be in good agreement with values from conventional electrochemical characterization.

Data & methodology:
The authors use an advanced and relatively new method, which is capable of providing detailed microscopic information. The approach is, as far as I can tell, valid, and the quality of data and presentation are very good. I do not, however, agree to the authors use of the term "operando". My understanding is that this is used within the community to describe conditions in which a device is operating, i.e. performing some function, e.g. a catalytic reaction. As far as I see, the present measurements should rather be described as "in situ".

Reply to the referee:

We are thankful to the referee for raising this important question, which is indeed a matter of debate in the scientific community since the early *operando/in situ* studies. We agree with the referee that the term '*operando*' is commonly used in the (electro)catalysis community to define a study were a physical system is probed while a process involving a chemical reaction and/or a catalytic cycle is occurring *during* the measurement itself.

In our work, we define '*operando*' as the experimental conditions where the electrified interface is probed as a function of the applied potential, i.e. *under polarization*. Therefore to avoid potential misunderstanding or confusion we replace "operando" with "*under polarization*".

The authors claim to have developed the "minimum width extrapolation method" (MWEM). To me it appears to consist of fitting two straight lines to the two halves of the data set. If there was any other development, this should be explained.

Reply to the referee:

The 'minimum width extrapolation method' (MWEM) consists of superimposing two fitted straight lines to the anodic and cathodic branch of the full width at half maximum (FWHM) values as a function of the applied potential. We agree with the referee that the naming could lead to misunderstandings. Accordingly, we have updated the main text avoiding the proposed name as follows:

"To extract the PZC from the spectroscopy data of both core levels, we have superimposed two fitted straight lines to the anodic and cathodic branch of the FWHM trend of the LPPy N 1s and LPW O 1s components as a function of the applied potential, as reported in Figure 2d. The PZC determined in this manner is equal to +160 mV and +141 mV by using the LPPy N 1s and LPW O 1s component, respectively, vs. +123 mV derived from the CV method."

In figure 4, the third row is said to report "the exponential potential drop within the EDL as function of both the concentration and the applied potential, directly derived from the comparison between the experimental data and the numerical simulations". How this derivation was made is unclear, and needs to be explained better.

Reply to the referee:

We have updated the main text explaining in detail how the derivation. Additional details have also been included in the SI section S7:

"…The simulations of the potential drop profiles, similarly to what has been experimentally performed, have been generated as a function of the applied potential for different electrolyte

concentrations (i.e. for different EDL thicknesses; more details are reported in SI, section S7). The comparison between the experimental and simulated FWHM provides the ability to directly access and visualize the potential drop within the EDL that leads to the observed spectral broadening. The resulting simulated exponential potential profiles are reported in Figure 4".

Appropriate use of statistics and treatment of uncertainties:
The discussion on different models for the EDL, as presented in fig 3, concludes that an exponential model describes the data best. This may well be true, but considering the error bars, at least the linear model cannot be excluded.

Reply to the referee:
The referee's concern reflects our motivation for presenting the chi square values, a statistically significant 'goodness of fit' parameter, to discern the potential drop model that best fits the data. We found this approach very beneficial, as it helped to objectively evaluate our data and models for all applied potentials and concentrations. From this analysis we were able to observe that the exponential model statistically and consistently provides the lowest chi square values, and thus provides the best fit and interpretation for the data.

However, we agree with the referee that with the current signal-to-noise level, we cannot definitively rule out the "linear model". Therefore, following the suggestions provided by the referee, we have updated the text as follows:
"The step and linear potential drop models were selected to test the fit to our data as well as our ability to distinguish the shape of the EDL structure, and thus do not represent a modern theory for the EDL structure. We utilized a statistically significant 'goodness of fit' parameter (chi-square) to discern which potential drop model (step, linear, and exponential) best fits the data. This approach provides the ability to objectively evaluate the agreement between the data and models for all applied potentials and electrolyte concentrations (i.e. EDL thicknesses)".

"…the comparison between the experimental data and the simulations based on the envelope function approach (see SI, section S7) have demonstrated that the best match is consistently achieved (for different electrolyte concentrations and using the spectral broadening of both LPPy N1s and LPW O1s core levels) when the potential drop is modeled with an exponential function, as predicted by the Gouy-Chapman-Stern model ((the chi-square values for the three different models and the investigated conditions are reported in Table S1, SI, section S7).). However, with the current signal-to-noise ratio, the definitive discrimination between an exponential and a linear shape for the potential drop is challenging to achieve".

"Future improvements of the experimental set-up will enhance spectral resolution and signal-to-noise ratio, which will improve our ability to discern the fine features of the potential drop functional dependency carried by the core level spectral broadening".

We have also reported the chi-square values for all the investigated conditions in the updated SI, see section S7, Table 1.

Conclusions:
The main conclusion, i.e. that the potential drop over the EDL can be probed with APXPS, seems robust and valid.
Clarity and context: The manuscript is well written, and the reasoning is easy to follow. The problem and the result are put into a larger context in a good way.

Reviewer #3 (Remarks to the Author):

The manuscript by Favaro et al. describes probing the electrochemical solid/liquid interface and the electrochemical double-layer (DL) both by electrochemical methods and by operando ambient pressure XPS (APXPS). This manuscript shows excellent new and original results, clearly demonstrating that the DL and the potential drop within the DL can be probed spectroscopically under operando electrochemical conditions overcoming many issues related to previous studies, where mainly the DL was simulated ex-situ in a UHV environment. I enjoyed reading this manuscript and there are not too many comments to be given. This is a very important work pathing the way to a significant better understanding of the DL and all the processes occurring at the solid-liquid interface...and I am surprised that this work is not published in one of the higher impact journals of the Nature family.

Reply to the referee:
We are deeply thankful to the referee for their appreciation of this work.

1. The main question related to this work relates to one important point: Is a charging of the electrolyte observed during the XPS measurements as e.g., observed within ionic liquids (see e.g., ECS Electrochemistry Letters 2(4)(2013) H13-H15) and how was it dealt with? Could charging effects also lead to distortions of the potential distribution within the DL resulting from core level shifts? As the ms is written, it appears this is not the case, but this important point needs to be clarified and the authors need to explain, why this may not be the case.

Reply to the referee:

We are thankful to the referee for raising this important point. As the referee already pointed out, we do not see binding energy shifts due to charging effects on our measurements. The figure reported below shows two different N and O 1s core levels acquired at the resting potential (open circuit potential, OCP) 3 hours apart with continuous X-ray exposure (i.e. at the beginning and at the end of an experiment where the EDL was probed as a function of the applied potential in an aqueous KOH 0.4 mM solution containing 1.0 M pyrazine). No BE shift over time is detected, nor a shift in the OCP (within the experimental resolution, about 10 mV). This example and other results from our experiments consistently show that charging is not an issue in our measurements.

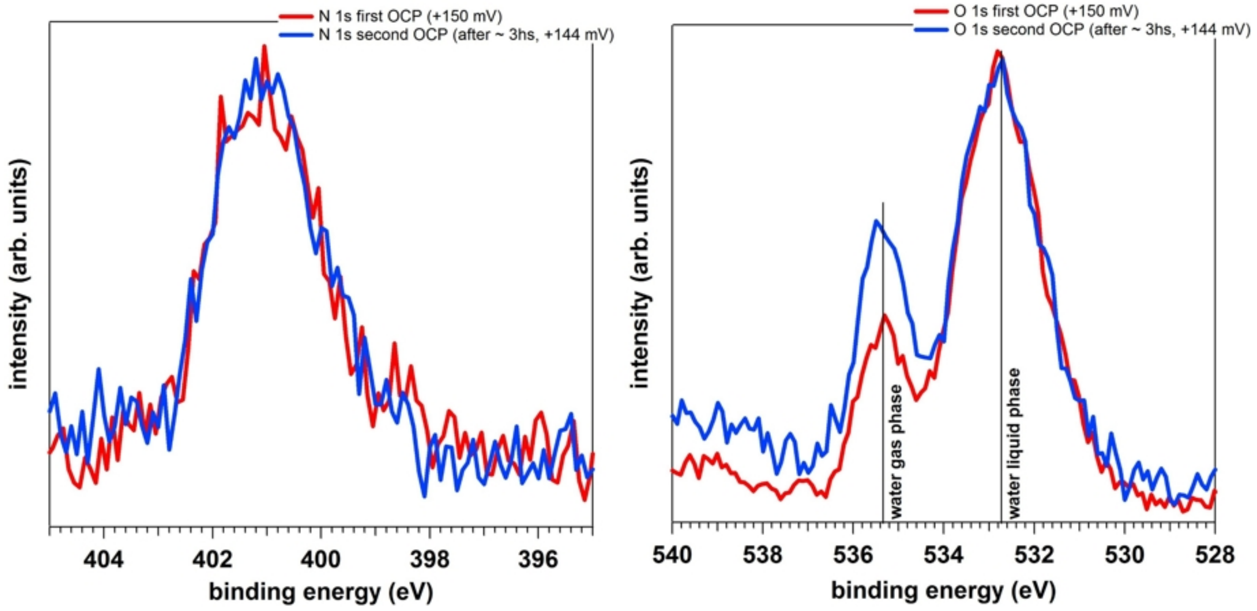

Beside the difference in the two electrolyte systems (aqueous vs. IL), one advantage of APXPS may help to explain the physical reasons: the fact that we performed the experiment at relatively high pressure (~ 18 torr) can alleviate the charging effect compared to UHV conditions. For example, APXPS was successfully used to study an insulator under ambient pressure conditions (F. Ogletree et al., *Rev. Sci. Inst.* **73**, 3872 (2002) - H. Bluhm, *J. Electron Spectrosc. Relat. Phenom.* **177**, 71 (2010)). As discussed in the previous literature, the high pressure around the sample helps in dissipating the samples charge accumulated by the absorbed X-ray dose. The irradiation of gas molecules (water) by the incident photons leads to the emission of photoelectrons, Auger and secondary electrons in the gas phase that can partially or even completely compensate the charge in the liquid phase.

In summary, we did not observe “charging” during our measurement. We believe that two aspects of the measurement (electrolyte difference and high water vapor pressure) contribute to the no “charging” of the electrolyte during our XPS experiment.

We added reference 48 (ECS Electrochemistry Letters 2. (2013) H13-H15) and the following in the updated text and SI to reflect this comment:

"Charging of the electrolyte [48] was not observed in this study during the APXPS measurements: Figure S15 (SI, section S10) shows both N 1s and O 1s core levels acquired at the OCP after 3 hours of X-ray exposure (i.e. initially collected at the beginning and again at the end of an experiment where the EDL was probed as a function of the applied potential in an aqueous KOH 0.4 mM solution containing 1.0 M pyrazine).The X-ray dose absorbed by the electrolyte does not lead to shift in the BE nor in the OCP values".

2. Besides the summary of the previous work on DLs, the authors should consider to also discuss specifically the older ex-situ UHV approaches and outline what additional information can be drawn from the operando XPS work (e.g., the work by Wagner & Ross, but also other summarized in ref. 18 of this ms.)

Reply to the referee:
We have updated the following text according to the suggestions provided by the referee:

"Since the pioneering studies of Kolb, Wagner, and Ross on emersed electrodes, several groups have employed X-ray photoelectron spectroscopy (XPS) measurements not under polarization conditions, showing that it is possible to study the specific adsorption of ions at the *Inner Helmholtz Plane* (IHP)".

"However, despite these initial fundamental studies on the EDL structure (18, 23-30), the information of the electrical potential profile at the solid/liquid *electrified* interface, particularly as a function of the applied potential and electrolyte concentration, was still eluding us. New experimental tools and approaches were needed to directly probe the potential drop in solution *under polarization* conditions".

3. This is mainly a comment towards the format: Even in the Letter size printout, figures 1, 2 and 3 are very difficult to read as everything is very small and barely visible (e.g., legends and descriptions). Figure 1 needs to be over both columns at least, otherwise information cannot be read.

Reply to the referee:
We have updated and improved the figures accordingly to the suggestions provided by the referee.

Reviewers' Comments:

Reviewer #1 (Remarks to the Author)

I found the authors answered all the questions raised by reviewers. I recommend the publication of manuscript.

Reviewer #2 (Remarks to the Author)

The authors have satisfactorily responded to my comments, and I have nothing further to add.

Reviewer #3 (Remarks to the Author)

Nice and imnportant work.
Should be published as is.

Reviewers' comments:

Reviewer #1 (Remarks to the Author):

I found the authors answered all the questions raised by reviewers. I recommend the publication of manuscript.

Reply to the referee:
We are glad we were able to answer all of the questions and are grateful to the referee for their appreciation of this work.

Reviewer #2 (Remarks to the Author):

The authors have satisfactorily responded to my comments, and I have nothing further to add.

Reply to the referee:
We are glad we were able to answer all of the questions and are grateful to the referee for their appreciation of this work.

Reviewer #3 (Remarks to the Author):

Nice and important work.
Should be published as is.

Reply to the referee:
We are glad we were able to answer all of the questions and are grateful to the referee for their appreciation of this work.
